# Ezrin drives adaptation of monocytes to the inflamed lung microenvironment

**DOI:** 10.1038/s41419-024-07255-8

**Published:** 2024-11-29

**Authors:** Ravindra Gudneppanavar, Caterina Di Pietro, Hasan H Öz, Ping-Xia Zhang, Ee-Chun Cheng, Pamela H. Huang, Toma Tebaldi, Giulia Biancon, Stephanie Halene, Adam D. Hoppe, Catherine Kim, Anjelica L. Gonzalez, Diane S. Krause, Marie E. Egan, Neetu Gupta, Thomas S. Murray, Emanuela M. Bruscia

**Affiliations:** 1 Department of Pediatrics, School of Medicine, Yale University, New Haven, CT USA; 2 Yale Stem Cell Center, School of Medicine, Yale University, New Haven, CT USA; 3 Department of Laboratory Medicine, School of Medicine, Yale University, New Haven, CT USA; 4 Department of Hematology, School of Medicine, Yale University, New Haven, CT USA; 5 Department of Cellular, Computational and Integrative Biology (CIBIO), University of Trento, Trento, Italy; 6 Department of Chemistry and Biochemistry, South Dakota State University, Brookings, SD USA; 7 Department of Biomedical Engineering, Yale University, New Haven, CT USA; 8 Department of Pathology, School of Medicine, Yale University, New Haven, CT USA; 9 Department of Cellular and Molecular Physiology, Yale University, New Haven, CT USA; 10 Department of Inflammation and Immunity, Cleveland Clinic Foundation, Cleveland, OH USA

**Keywords:** Phagocytes, Extracellular matrix

## Abstract

Ezrin, an actin-binding protein, orchestrates the organization of the cortical cytoskeleton and plasma membrane during cell migration, adhesion, and proliferation. Its role in monocytes/macrophages (MΦs) is less understood. Here, we used a monocyte/MΦ-specific ezrin knock-out mouse model to investigate the contribution of ezrin to monocyte recruitment and adaptation to the lung extracellular matrix (ECM) in response to lipopolysaccharide (LPS). Our study revealed that LPS induces ezrin expression in monocytes/MΦs and is essential for monocytes to adhere to lung ECM, proliferate, and differentiate into tissue-resident interstitial MΦs. Mechanistically, the loss of ezrin in monocytes disrupts activation of focal adhesion kinase and AKT serine-threonine protein kinase signaling, essential for lung-recruited monocytes and monocyte-derived MΦs to adhere to the ECM, proliferate, and survive. In summary, our data show that ezrin plays a role beyond structural cellular support, influencing diverse monocytes/MΦ processes and signaling pathways during inflammation, facilitating their differentiation into tissue-resident macrophages.

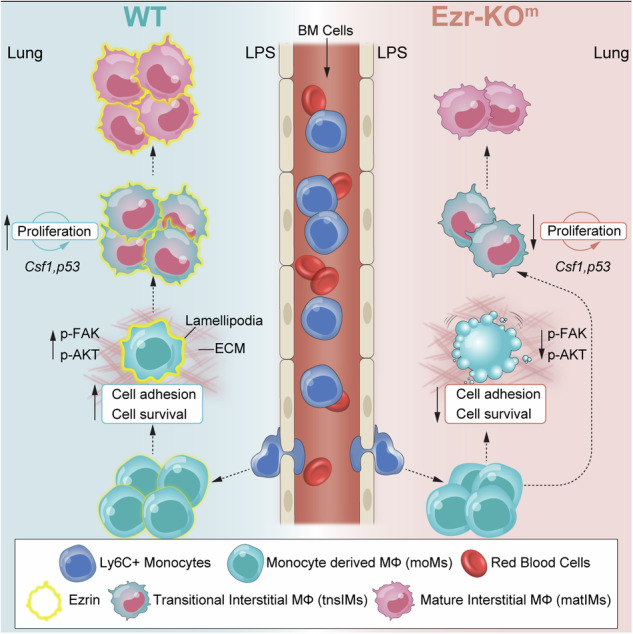

## Introduction

Plasma membrane (PM) rearrangement is a feature of monocyte/macrophage (MΦ) activation and is required for a controlled and efficient cell response to infection. Ezrin, a member of the ezrin-radixin-moesin (ERM) protein family, is an actin-binding protein that is required for the cell cortical cytoskeleton and PM organization of the cell. In response to external stimuli, kinases (e.g., LOK, ROCK, PKC) activate ezrin by phosphorylating its C-terminal domain at Thr-567 [1]. This action unmasks ezrin’s binding sites for F-actin at the C-terminus and for the inner leaf of the PM at the N-terminus. Using these binding sites, ezrin anchors the F-actin bundles to the PM through interactions with phosphatidylinositol 4,5-bisphosphate (PIP2) and specific PM proteins, such as CD44, ICAM1, EBP50 and EGFR [1–4]. Thus, ezrin contributes to the lateral organization of the PM in response to kinase activation, thereby playing a role in cellular processes such as proliferation, adhesion, signaling, migration, and polarization [3, 4].

Extensive studies in epithelial cells show that ezrin controls cell polarity and migration. However, the role of ezrin in immune cells is less appreciated, with most research focused on moesin [5]. Studies that address the specific role of ezrin in immune cells have revealed cell type-specific functions. In lymphocytes, ezrin organizes cortical actin below the uropod membrane to control adhesion, polarity, and migration [4, 6–8]; it facilitates the clustering of B and T cell receptors, thereby modulating downstream signaling and lymphocyte activation [9–11] and mediates T cell immunological synapse formation [9, 12]. Moreover, a homozygous mutation of the ezrin gene was identified in a patient with a B-cell deficiency [13]. We and others have demonstrated the expression of ezrin in myeloid cells during the activation process [14–16]. In both MΦs and neutrophils, ezrin phosphorylation is required for efficient phagocytosis and phagolysosome formation and fusion [15, 16]. In vitro, altered levels and distribution of ezrin in MΦs led to a blunted PI3K/AKT signaling in response to TLR4 activation and impaired phagocytosis, culminating in increased production of pro-inflammatory cytokines [14].

We previously reported that the level of ezrin is reduced in the MΦs of individuals with cystic fibrosis (CF) [14], a disease characterized by lung hyperinflammation, and chronic infection initiated by airway dehydration and compromised mucociliary clearance [17, 18]. The lungs of people affected with CF have an increased abundance of classical monocytes recruited from the circulation [19, 20]. This imbalance is a common feature in many lung diseases, characterized by long-lasting and unresolved inflammation in response to infection or tissue damage [21, 22]. In the initial phases of pulmonary infection, the lungs are rapidly populated by waves of Ly6C^+^ classical monocytes recruited from the bloodstream, to help initiate the pro-inflammatory response. Over time, Ly6C^+^ monocytes undergo proliferation and acquire a tissue-resident MΦ phenotype (monocyte-derived MΦs or moMs) influenced by the lung microenvironment (interstitium or alveoli). Once extravasated into the lung tissues, Ly6C^+^ monocytes proliferate locally, in a colony-stimulating factor 1 (Csf1) receptor-dependent manner, and this proliferative stage precedes their differentiation into mature interstitial macrophages (IMs) [23]. IMs are known for their immunomodulatory functions [24] and are involved in pathological lung processes when dysregulated, such as in idiopathic pulmonary fibrosis [25] and asthma [24]. Therefore, the dysregulated transition of Ly6C^+^ monocytes to mature IM phenotype could potentially contribute to the pathogenesis of many pulmonary diseases, including CF [19].

Upon inducing acute pulmonary inflammation in a mouse model in which ezrin is selectively knocked out (KO) in monocyte/MΦ, our study uniquely highlights the pivotal role of ezrin for activation of the focal adhesion associated kinase (FAK)/protein kinase B (AKT) axis signaling in lung-recruited monocyte/MΦ, thus promoting their cell adhesion to the lung extracellular matrix (ECM), survival, proliferation, and differentiation. Taken together, these findings underscore the crucial involvement of ezrin in mediating the adaptation of blood-recruited monocytes to the lung tissue microenvironment during inflammation.

## Results

### Ezrin is induced in monocytes/MΦs in response to LPS and is efficiently knocked down in monocytes/MΦs of *Ez*^fl/fl^ × *Cx3cr1*^Cre+^ mice

To investigate the role of ezrin during monocytes/MΦs activation, we developed a novel mouse model with ezrin knocked out (KO) specifically in monocytes/MΦs by crossing the B6.*Ez*^fl/fl^ mouse [26, 27] with the B6.*Cx3cr1*^tm1.1(cre)Jung/J^ mouse line (*Ez*^fl/fl^ × *Cx3cr1*^Cre+^; hereafter *Ezr*-KO^m^) (Supplementary Fig. S1A) [28] for comparison with wild-type mice (*Ez*^+/+^ × *Cx3cr1*^Cre+^; hereafter WT). Using a B6.129P2(Cg)-*Cx3cr1*^tm1Litt/J^ (*Cx3cr1*^GFP^) mouse model [29], we assessed the activity of the *Cx3cr1* promoter in immune cells, specifically examining GFP expression levels. We confirmed a robust expression of the *Cx3cr1*-Cre GFP in the lung MΦs such as mo-AMs, Ly6C^+^ monocyte-derived MΦs (moMs), IMs, Ly6C^−^ moMs (Supplementary Fig. S1B), as well as in blood and bone marrow (BM) Ly6C^+^ and Ly6C^−^ monocytes (Supplementary Fig. S1C, D). We did not observe *Cx3cr1*-Cre GFP expression in alveolar macrophages (AMs). However, in AMs from the *Ezr*-KO^m^ mice, we observed a decrease in ezrin mRNA expression (Supplementary Fig. S1E), supporting the notion that *Cx3cr1*-Cre is expressed in the early stages of AM differentiation, thus driving the ezrin knockout in this population as well, consistent with previous studies [28]. Importantly, the *Cx3cr1*-Cre GFP is not expressed in granulocytes, although a low level of expression can be seen in classical dendritic cells (cDCs), eosinophils (Eos), and natural killers (NK) cells (Supplementary Fig. S1B). These data validated this mouse model of reduced ezrin expression to investigate the role of ezrin expression in monocyte/MΦs populations.

**Fig. 1:**
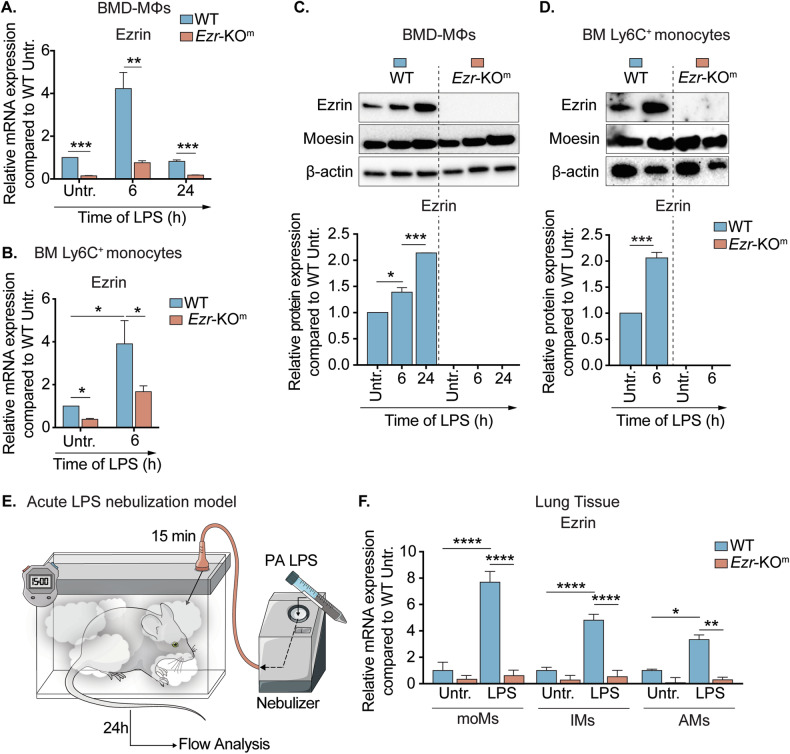
Ezrin is induced in monocytes and MΦs in response to LPS. Quantitative PCR (qPCR) for ezrin in WT and *Ezr*-KO^m^ mouse bone marrow-derived macrophages (BMD-MΦs) (**A**) and primary bone marrow (BM) monocytes (**B**), untreated or treated with LPS for 6 h and 24 h. The relative mRNA expression of ezrin was normalized to *STX5a* and compared to WT untreated (Untr). Western blot (WB) and densitometric analysis for ezrin and moesin in mouse BMD-MΦs (**C**) and BM monocytes (**D**), untreated or treated with LPS for 6 h and 24 h. Protein fold increase was normalized to β-actin and relative to untreated cells. **E** Cartoon representation of the in vivo model of *Pseudomonas aeruginosa*—lipopolysaccharide (PA-LPS) nebulization. **F** qPCR of ezrin in murine WT and *Ezr*-KO^m^ monocyte-derived MΦs (moMs), interstitial MΦs (IMs) and alveolar MΦs (AMs), sorted from lung tissues of untreated or LPS treated mice. The relative ezrin mRNA expression was normalized to *STX5a* and the WT Untr., distinctly for each phenotype. Data are represented as mean ± SEM from three independent experiments with three or more mice per genotype. Statistical analysis was performed using one-way ANOVA or Tukey’s test for multiple comparisons between the genotype and treatment conditions. *p < 0.05, **p < 0.01 and ***p < 0.001. See also Supplementary Fig. S2.

To investigate the transcriptional and translational expression of ezrin in WT mice, we assessed primary murine bone marrow-derived (BMD) MΦs and primary bone marrow (BM) Ly6C^+^ monocytes cultured on a collagen surface at steady-state and after exposure to lipopolysaccharide (LPS) from *Pseudomonas aeruginosa* (PA) [30, 31]. *Ezr*-KO^m^ mice were used as controls. We selected PA because it frequently colonizes the lungs of patients with CF (pwCF) and is associated with severe lung disease in CF [32]. In WT mice, ezrin’s mRNA expression is initially expressed at low basal levels in both BMD-MΦs and BM Ly6C^+^ monocytes. After 6 h of LPS exposure, ezrin mRNA expression increases four-fold (Fig. 1A, B), accompanied by a rise in protein levels that persists for up to 24 hours after exposure to LPS (Fig. 1C, D). In contrast, the expression of the other ERM protein family members, moesin and radixin, is not induced by LPS (Fig. 1C, D and Supplementary Fig. S2A). We also observed that ezrin induction is more robust in response to LPS compared with other inflammatory stimuli such as type-I and type-II interferons (Supplementary Fig. S2B), supporting a TLR-mediated induction [14]. As expected, ezrin mRNA expression and protein levels were significantly downregulated in BMD-MΦs and BM primary monocytes in *Ezr*-KO^m^ mice compared with WT mice (Fig. 1A–D), during both the steady state and in response to LPS. Additionally, radixin and moesin expression remained unchanged among WT and *Ezr*-KO^m^ cells (Supplementary Fig. S2A), demonstrating a lack of other ERM protein’s compensatory effect in the *Ezr*-KO^m^ background.

We next examined lung monocytes/MΦs isolated from WT and *Ezr*-KO^m^ mice using fluorescence-activated cell sorting (FACS) using a gold-standard flow cytometry gating strategy specific to identify lung monocytes/MΦs populations in mice (Supplementary Fig. S2C) [19, 33, 34]. LPS aerosolization induced abundant lung recruitment of Ly6C^+^ monocytes that over time differentiate into different monocyte/MΦ lung populations, including Ly6C^+^ MHCII^-^ monocyte-derived MΦs (moMs) and Ly6C^-^ MHCII^+^ interstitial MΦs (mature IMs) [19, 35]. Consistently, in WT mice treated with LPS, we observed a dramatic increase in lung Ly6C^+^ moMs and IMs between 6 h and 24 h after LPS nebulization, while the number of AMs showed minimal changes (Supplementary Fig. S2D). At a steady state, ezrin expression remained low in all assessed MΦs populations (moMs, IMs, and AMs) sorted from WT mouse lung tissues. However, ezrin expression increased in all these monocyte/MΦ populations of WT mice exposed to LPS (Fig. 1F), with no alterations observed in moesin and radixin levels in moMs and IMs (Fig. S2E), consistent with in vitro findings. Notably, the highest induction of ezrin in WT was observed in moMs and IMs, compared with AMs (Fig. 1F). As expected, ezrin mRNA expressions were significantly downregulated in sorted primary lung moMs, IMs, and AMs from the *Ezr*-KO^m^ mice compared with WT mice (Fig. 1F), at the steady state and in response to LPS. In addition, radixin and moesin expression remained unchanged in *Ezr*-KO^m^ moMs and IMs (Supplementary Fig. S2E). Altogether, our data show that ezrin expression is induced by LPS, suggesting that it plays a distinctive and non-redundant role compared to other ERM proteins in the activation of monocytes/MΦs within lung tissues in response to infections. Additionally, our robust genetic mouse model allows us to address the specific role of ezrin in lung monocytes/MΦs in response to inflammation.

### The loss of ezrin impacts the number of lung monocyte-derived MΦs in response to LPS

**Fig. 2:**
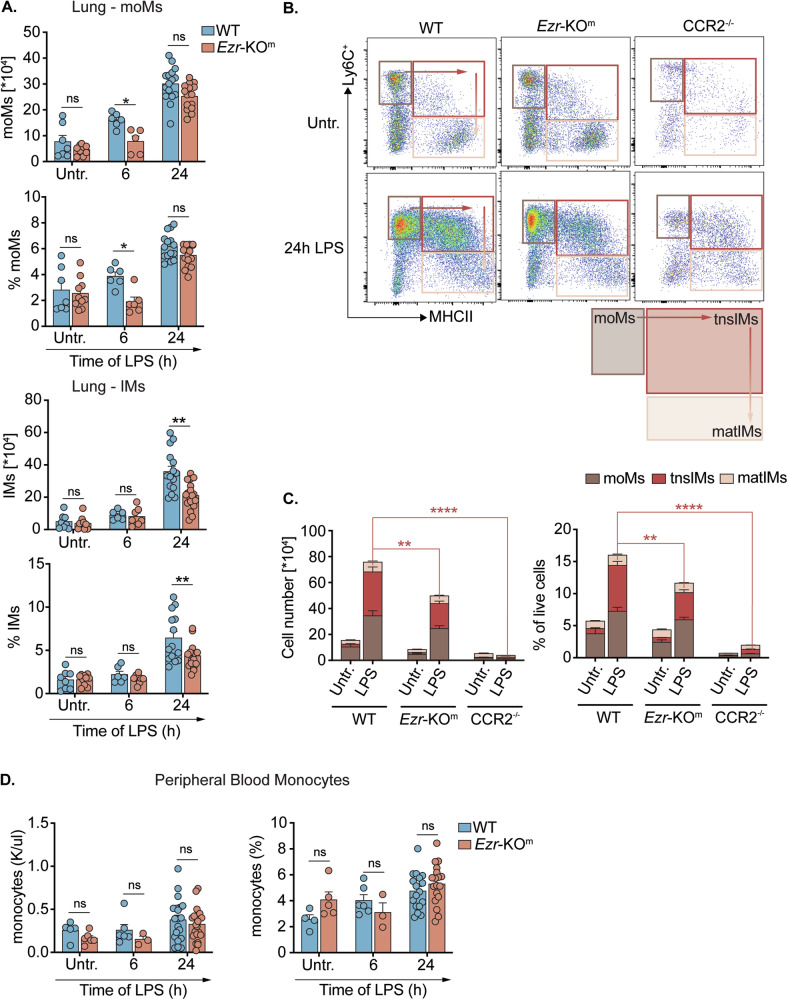
The loss of ezrin impacts the number of lung monocyte-derived IMs in response to LPS. WT and *Ezr*-KO^m^ mice were nebulized with LPS (12.5 mg/5 ml for 15 min). The mice were sacrificed post-24 h of LPS and collected blood, BALF and lung tissue samples for flow cytometry and RNA analysis. **A** Numbers and percentages of lung moMs and IMs in lung tissue homogenates (inferior lobe) were quantified by sequential gating strategy from flow cytometry. Cell numbers were calculated from the percentage of viable cells multiplied by the total cell count in the inferior lung lobe. The gating strategy is described in Fig. S2C. **B** Flow cytometry-based dot plot representation of moMs, transitional IMs (tnsIMs) and mature IMs (matIMs) populations in WT, *Ezr*-KO^m^ and *CCR2*-KO mice, untreated or treated with LPS. **C** Numbers and percentages of moMs, tnsIMs and matIMs (quantification of (**C**)) in the lung (inferior lobe) tissue, untreated or treated with LPS. Quantification was performed as in (**B**). **D** Number (left) and percentage (right) of monocytes in the peripheral blood. Data are represented as mean ± SEM from three independent experiments with three or more mice per genotype. Each dot represents a biological replicate. Statistical analysis was performed using one-way ANOVA or Tukey’s test for multiple comparisons between the genotype and treatment conditions. *p < 0.05, **p < 0.01 and ns non significant. See also Supplementary Figs. S3–5.

We next investigated the role of ezrin in the dynamic changes of lung monocyte and MΦ populations during the inflammatory response in both WT and *Ezr*-KO^m^ mice following LPS nebulization (Fig. 1E). At steady state, the number of tissue resident lung monocytes/MΦs did not differ between genotypes (Fig. 2A). In lung tissues, we observed a slight decrease in both the number and percentage of moMs in *Ezr*-KO^m^ mice compared with WT at 6h-post LPS. However, the difference between the two mouse strains faded at 24 h-post LPS, suggesting that ezrin plays a marginal role in recruiting moMs from the bloodstream into the lung parenchyma. In contrast, LPS-treated *Ezr*-KO^m^ mice show a reduction of lung MΦs compared with WT mice (Supplementary Fig. S3A, B), which we established to be caused by a robust impairment in the LPS-driven expansion of IMs (Fig. 2A) relative to WT mice. The absence of ezrin did not affect the counts of lung AMs, Ly6C^−^ monocytes, neutrophils, DCs, or eosinophils in response to LPS (Supplementary Fig. S4A). These data collectively highlight the critical role of ezrin induction in the expansion of monocyte-derived lung IMs in response to LPS.

To further distinguish IMs differentiating from Ly6C^+^ moMs within the lung parenchyma during inflammation, we gated the IMs populations based on Ly6C and MHCII markers expression [23, 36, 37]. This allowed us to identify two distinct IM populations: Ly6C^+^ MHCII^+^ IMs (transitional IMs; referred as tnsIMs) and Ly6C^−^ MHCII^+^ IMs (differentiated mature IMs with a tissue-resident phenotype; hereafter matIMs) (Fig. 2B) [23, 35]. Under steady state conditions, the lungs were predominantly populated by tissue-resident IMs, with no differences between WT and *Ezr*-KO^m^ mice. In response to LPS, both the numbers and percentages of moMs, tnsIMs, and matIMs in WT and *Ezr*-KO^m^ mice increased in a CCR2-dependent manner (Fig. 2B, C). This suggests that the expansion of tnsIMs and matIMs populations following LPS stimulation originated from recruited Ly6C^+^ CCR2^+^ monocytes. Notably, the loss of ezrin specifically impacted the number and percentage of tnsIMs subpopulation post-LPS induction (Fig. 2B, C). Thus, our data further indicate the significance of ezrin induction for the adaptation of moMs and the transition and expansion of tnsIMs toward differentiation into matIMs [23] within the lung parenchyma.

We also assessed whether loss of ezrin impacts the number/percentage of circulating monocytes. Both at steady state (Untr.) and in response to LPS stimulation WT and *Ezr*-KO^m^ mice showed no significant differences in the number of monocytes in the peripheral blood (Fig. 2D), suggesting that ezrin does not affect the extravasation of monocytes from the bone marrow. In addition, we observed no difference in complete blood count (CBC) measurements of total white blood cells (WBCs), neutrophils, lymphoid cells, eosinophils, and basophils between *Ezr*-KO^m^ and WT mice at 6 h and 24 h-post LPS (Supplementary Fig. S4B). Taken together, these data suggest that ezrin is primarily necessary when circulating monocytes extravasate into the lungs and become activated by the inflamed microenvironment.

Finally, we assessed the impact of ezrin loss in monocytes/MΦs on lung inflammation in *Ezr*-KO^m^ mice at 6 h and 24-post LPS, compared with WT mice. We observed a gradual increase in total protein levels (Supplementary Fig. S5A) and an early (6 h) high number of neutrophils (Supplementary Fig. S5B) in the bronchoalveolar-lavage fluid (BALF) of *Ezr*-KO^m^ mice in response to LPS compared with WT, suggesting increased lung inflammation in *Ezr*-KO^m^ mice. Analysis of pro-inflammatory cytokines expression in lung tissues revealed increased mRNA levels only for *Cxcl1*, post 6 h LPS in *Ezr*-KO^m^ mice, which accounts for neutrophil recruitment in the alveolar space (Supplementary Fig. S5B) and no statistical differences were observed for other cytokines (Supplementary Fig. S5C). However, when we examined the expression of these pro-inflammatory cytokines in sorted lung MΦs (moMs and IMs) from LPS-treated WT and *Ezr*-KO^m^ mice, we observed elevated expression of cytokine *TNF-α*, as well as chemokines *Cxcl1* and *Cxcl2*, in LPS-induced *Ezr*-KO^m^ IMs, but not moMs (Supplementary Fig. S5D, the data represents technical replicates of lung-sorted cells pooled from three mice per group). Altogether these data suggest that ezrin deficiency causes a specific impact on the production of pro-inflammatory mediators and neutrophil chemoattractants in IMs during the early stage of immune response to LPS.

### LPS-treated WT and *Ezr*-KO^m^ lung MΦs have an altered expression profile consistent with a lack of differentiation toward a tissue-resident macrophage phenotype

**Fig. 3:**
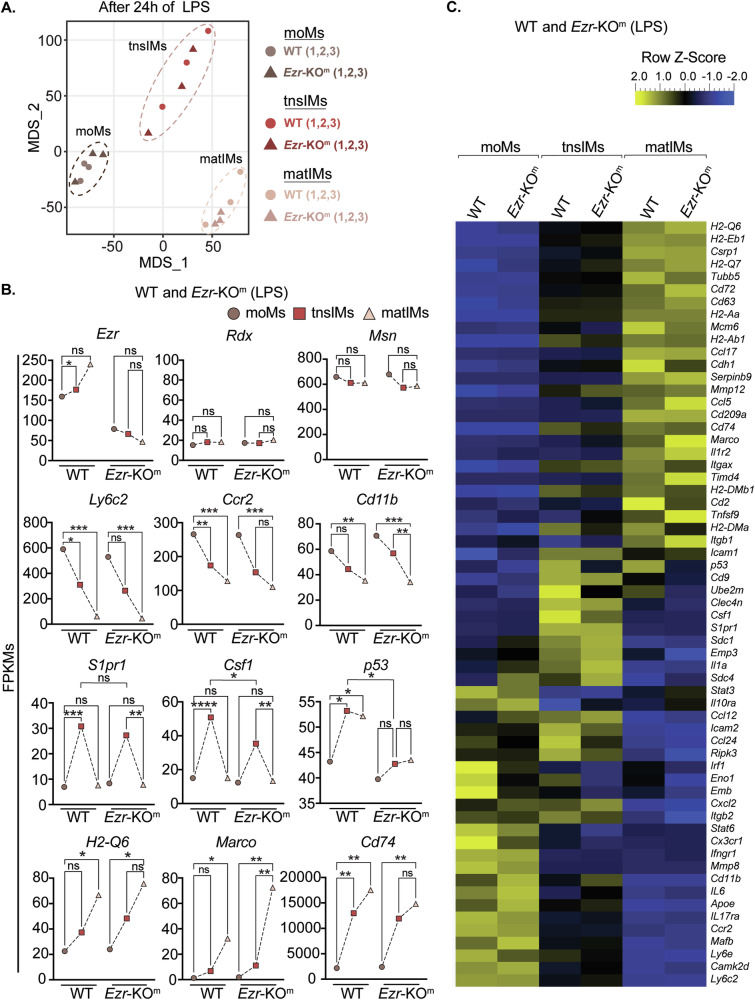
LPS-treated WT and *Ezr*-KO^m^ lung MΦs have an altered expression profile consistent with a lack of differentiation toward a tissue-resident macrophage phenotype. **A** Multidimensional scaling (MDS) plot depicting the distinct clusters of moMs, tnsIMs and matIMs in WT and *Ezr*-KO^m^ mice based on RNA expression profiles. **B** Comparative expression of cellular markers and their transition gene expression profiles in moMs, tnsIMs and matIMs between WT and *Ezr*-KO^m^ mice treated with LPS. **C** Hierarchical clustered heatmap depicting RNA expression profiles of differentially expressed genes (DEGs) comparing moMs, tnsIMs and matIMs in WT and *Ezr*-KO^m^ mice treated with LPS. Each tile represents the triplicate average of normalized fragments per kilobase of transcript per million mapped (FPKM) reads and colored according to gene-specific z-scores. Data were generated from three mice per genotype. Data are represented as median from three biological replicates. Statistical analysis was performed using a t-test (non-parametric) between the subpopulations. *p < 0.05, **p < 0.01, ***p < 0.001 and ns non significant. See also Supplementary Fig. S6.

To investigate ezrin’s contribution to the function of Ly6C^+^ monocytes, and their progeny in response to LPS, we sorted moMs, tnsIMs, and matIMs populations from the lung of WT and *Ezr*-KO^m^ mice 24 h after LPS nebulization and analyzed the transcriptional expression profiles by bulk RNA sequencing (RNA-seq). Consistent with the sorted macrophage populations, the principal component analysis (PCA) visualized distinct groups of MΦ phenotypes, among which tnsIMs clustered between moMs and matIMs (Fig. 3A) in both WT and *Ezr*-KO^m^ mice. All the assessed *Ezr*-KO^m^ MΦ populations revealed a similar number of differentially expressed genes (DEGs) compared with WT (Supplementary Fig. S6A). Among the 14,688 quantified gene expression profiles within WT and *Ezr*-KO^m^ samples, we identified DEGs specifically expressed in moMs, tnsIMs and matIMs (Supplementary Fig. S6A). We observed a gradual increase in the transcriptional expression of ezrin within WT moMs, tnsIMs and matIMs, whereas expressions of moesin and radixin remained consistent across the three populations (Fig. 3B). We also confirmed that ezrin transcript levels were significantly downregulated in moMs, tnsIMs and matIMs of *Ezr*-KO^m^ mice, while the expression levels of radixin and moesin remained unchanged compared to WT mice (Fig. 3B).

**Fig. 4:**
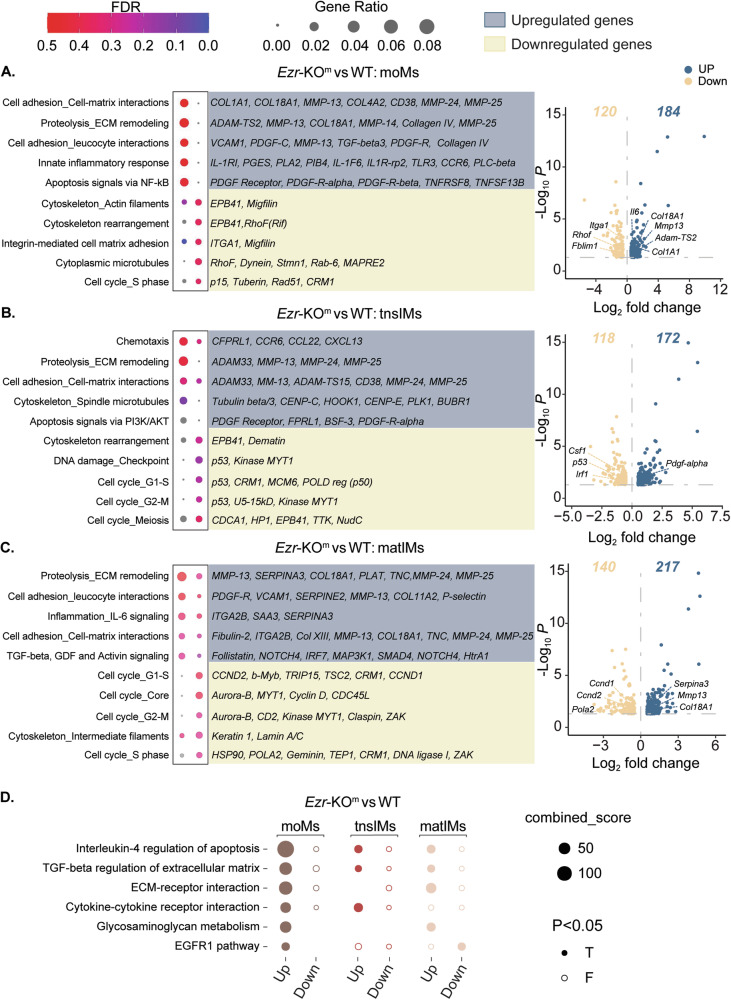
MetaCore enrichment pathway analysis of DEGs in LPS-treated WT and ezrin-KO moMs, tnsMs and matMs. **A**–**C** MetaCore enrichment pathway analysis and volcano plots of differentially expressed genes (DEGs) in *Ezr*-KO^m^ vs WT moMs (**A**), tnsIMs (**B**) and matIMs (**C**) populations treated with LPS. Dot plots show upregulated or downregulated pathways in *Ezr*-KO^m^ cell phenotypes and their associated genes. **D** Bioplanet pathway analysis of differentially expressed genes in *Ezr*-KO^m^ vs WT moMs, tnsIMs and matIMs populations treated with LPS. Node size: odds ratio associated with enrichment; p-value for Fisher’s exact test. Data were generated from three mice per genotype. See also Supplementary Fig. S6.

WT and *Ezr*-KO^m^ moMs exhibited the highest expression of receptors such as *Ly6c2, Ccr2*, and *Cx3cr1* and integrins (*Cd11b*) compared to tnsIMs and matIMs, consistent with their blood origin [38]. The expression of these monocyte-specific genes decreased as cells transitioned to tnsIMs and matIMs (Fig. 3B, C). However, no differences were observed between WT and *Ezr*-KO^m^ moMs. Focusing on DEGs between *Ezr*-KO^m^ and WT moMs, MetaCore enrichment analysis (MEA) revealed that *Ezr*-KO^m^ moMs exhibited higher expression of genes related to ECM remodeling (*ADAM-TS2*, *Col1A1*, *Col18A1*, *MMP13*) and increased expression of TGFβ3 ligand, which suggests that loss of ezrin in these cells may potentially contribute to an abnormal tissue remodeling process (Fig. 4A, D). *Ezr*-KO^m^ moMs also had upregulated DEGs related to the inflammatory response (*Il6*), while genes associated with cell adhesion (focal adhesion) and cytoskeleton rearrangement (*Itga1*, *Fblim1, Rhof*) were downregulated (Fig. 4A, D and Supplementary Fig. S6B), suggesting a defect in cell interaction with the lung ECM.

The tnsIMs in WT mice expressed a distinct set of genes related to actin cytoskeleton rearrangement (*Csf1* [39, 40], *S1pr1*), proliferation (*Csf1, Irf1*), and differentiation (*Csf1, p53)* (Fig. 3B, C). These findings align with recent data [23] showing that moMs acquire a proliferative phenotype (tnsIMs) and respond to MΦ differentiation factors (e.g., *Csf1*). Notably, in *Ezr*-KO^m^ tnsIMs *Csf1, Irf1* and *p53* levels were significantly downregulated compared to WT (Fig. 3B, C). MEA of *Ezr*-KO^m^ tnsIMs confirmed increased expression of genes related to apoptotic responses (*Pdgf-α*), and decreased expression of key genes regulating the cell cycle (*p53, p50, Myt1*) (Fig. 4B). Altogether, these data suggest that ezrin-deficient tnsIMs exhibit altered cytoskeletal rearrangement, defects in cell division and DNA replication, and increased cell death, leading to the loss of the proliferative pool of tnsIMs needed for the transition to differentiated matIMs. This may account for the reduced number of MΦs observed in the lung parenchyma of *Ezr*-KO^m^ mice compared to WT in response to LPS (Fig. 2 and Supplementary Fig. S3).

Compared with moMs and tnsIMs, and in line with the phenotype of fully differentiated IMs [23], WT and *Ezr*-KO^m^ matIMs expressed higher levels of genes related to ECM adhesion and remodeling (*Itgb1, Mmp12*), MHC-II associated genes (*H2-Q6/7, H2-Aa, CD72, CD74*) and scavenger receptor genes (*Marco*) (Fig. 3B, C). Further, MEA of *Ezr*-KO^m^ matIMs had both upregulation of ECM proteolytic enzymes (*Mmp-13, Serpina3, Col18A1*) along with downregulation of cell cycle (*Ccnd1, Ccnd2, Pola2*) and cell adhesion genes compared to WT matIMs (Fig. 4C and Supplementary Fig. S6B). Thus, *Ezr*-KO^m^ matIMs may have increased lung ECM remodeling activities coupled with decreased cell adhesion and dividing capabilities.

Interestingly, the three populations also exhibited distinct cytokine and chemokine expression patterns. WT moMs were identified as the primary producers of the pro-inflammatory cytokine *Il6*, and tnsIMs displayed elevated levels of the neutrophil chemoattractant *Cxcl2* and the pro-inflammatory cytokine *Il1α* (Fig. 3C). The matIMs population were characterized by the expression of cytokines associated with chemoattraction of T cells (*Ccl5*) (Fig. 3C). Ezrin loss in moMs and tnsIMs further enhanced the expression of *Il6*, *Cxcl2* and *Il1α* in response to LPS (Fig. 3C and Supplementary Fig. S5D). All together these data suggest that loss of ezrin negatively impacts the adaptation of moMs to the lung parenchyma, and transition to a proliferative pool of IMs in response to LPS.

### Monocyte/MΦs lacking ezrin exhibit proliferation defects in response to LPS

**Fig. 5:**
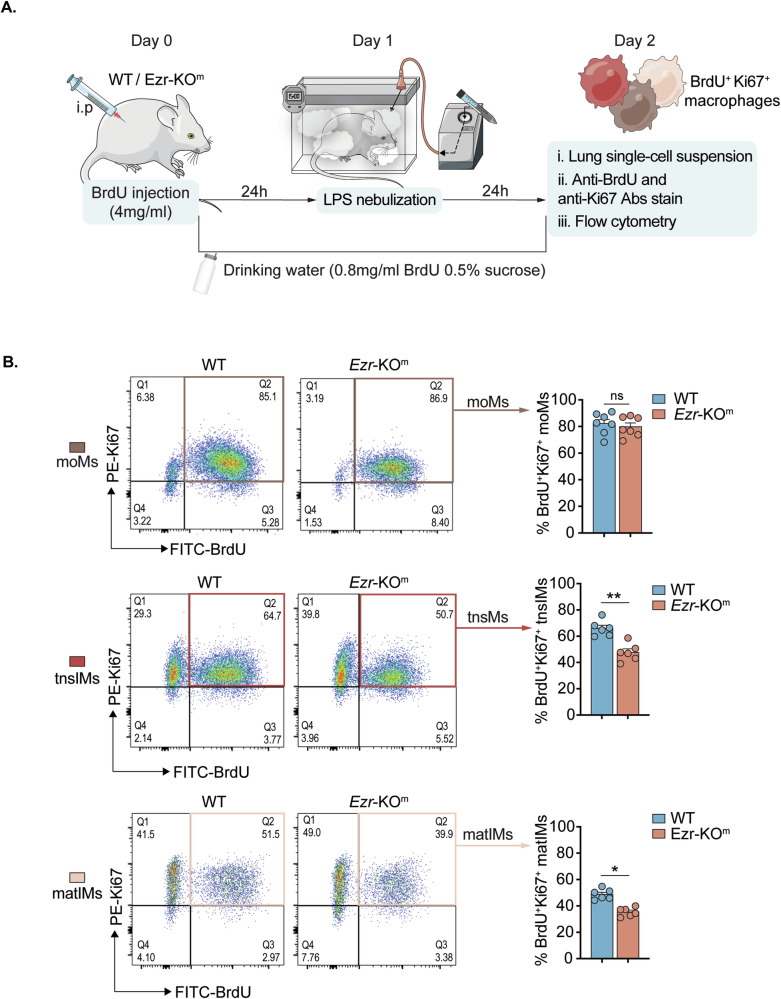
Monocyte/MΦs lacking ezrin exhibit proliferation defects in response to LPS. **A** Schematic of in vivo proliferation assay model. WT and *Ezr*-KO^m^ mice were administered with BrdU via intraperitoneal injection starting 24 h before the LPS nebulization. BrdU concentration was maintained in their drinking water throughout the experiment. **B** Flow cytometry analysis of moMs, tnsIMs and matIMs populations, co-stained with anti-BrdU (FITC) and anti-Ki67 (PE) antibodies, in WT and *Ezr*-KO^m^ mice treated with LPS. Bar graphs represent their corresponding percentages of BrdU^+^ Ki67^+^ cells. Data are represented as mean ± SEM from two independent experiments with three or more mice per genotype per experiment. Each dot represents a biological replicate. Statistical analysis was performed using one-way ANOVA or Tukey’s test for multiple comparisons between the genotype and treatment conditions. *p < 0.05, **p < 0.01 and ns non significant. See also Supplementary Fig. S7.

Based on the RNA-seq analysis, we identified the downregulation of pathways that might impair the proliferation of tnsIMs and matIMs in *Ezr*-KO^m^ mice compared with WT mice (Fig. 4B, C). Thus, we functionally assessed the impact of ezrin on lung MΦ proliferation in response to LPS stimulation using an in vivo BrdU and Ki67 proliferation assay. WT and *Ezr*-KO^m^ mice were pretreated with BrdU before nebulizing with LPS, and BrdU and Ki67 stained single-cell populations from lung tissues were analyzed (Fig. 5A). LPS-induced WT moMs, tnsIMs and matIMs showed positive staining for the distinct BrdU^+^ Ki67^+^ population, indicating their proliferation status during the inflammatory response (Fig. 5B). The Ki67^+^ mean fluorescence intensities (MFI) in tnsIMs and matIMs were higher than moMs (Supplementary Fig. S7A), suggesting that in the condition of an inflammatory response proliferation is enhanced in tnsIMs and matIMs compared to moMs in WT mice. In contrast, we observed that *Ezr*-KO^m^ mice, compared with WT mice, have a significant reduction in the percentages of BrdU^+^ Ki67^+^ tnsIMs and matIMs (but not moMs) in response to LPS (Fig. 5B), suggesting a defect in the S phase of cell cycle and DNA replication in these populations. Additionally, we observed that Ki67^+^ MFI values were significantly reduced only in tnsIMs, but not in moMs and matIMs, when comparing WT and *Ezr*-KO^m^ mice (Supplementary Fig. S7B), suggesting that tnsIMs possess impairment in cell cycle phases specifically within G1 to M. Although proliferation impairment was observed in both tnsIMs and matIMs of *Ezr*-KO^m^ mice, tnsIMs exhibited defects across all cell cycle phases, including impaired DNA replication. Thus, we have functionally validated the RNA-Seq data, confirming that the loss of ezrin affects the proliferation efficiency of tnsIMs within the lung parenchyma, which aligns with the impaired expansion of the tnsIMs observed in *Ezr*-KO^m^ lungs (Fig. 2B, C), and their pivotal proliferative role during the repopulation of the lung niche [23].

### Ezrin is required for efficient monocyte/MΦ filipodia formation and cell spreading during activation with LPS

**Fig. 6:**
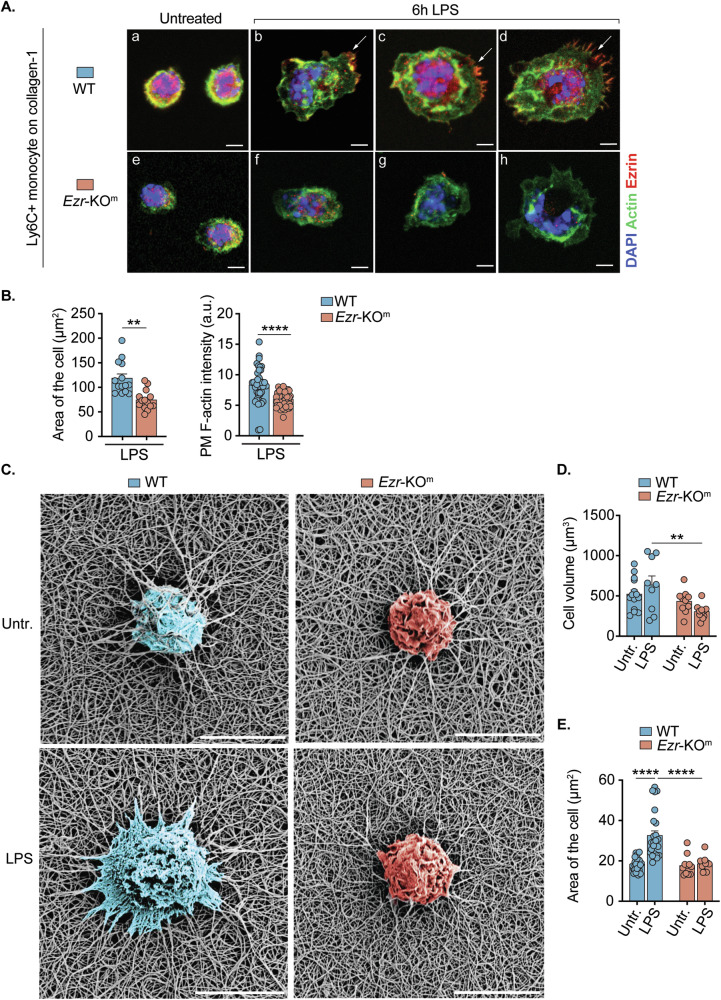
Ezrin is required for efficient monocyte/MΦ filipodia formation and cell spreading during activation with LPS. **A** Primary BM monocytes were isolated from murine WT and *Ezr*-KO^m^ and treated with LPS for 6 h on a collagen-coated surface. Representative immunofluorescence (IF) showing murine WT and *Ezr*-KO^m^ primary BM monocytes, untreated (**A**, **E**) or treated with LPS for 6 h (**B**–**D** and **F**–**H**). DAPI represents nucleus, Green represents actin and Red represents Ezrin staining. Scale bar = 2 μm. **B** Quantification of the area of the cells and plasma membrane (PM) F-actin intensity in murine WT and *Ezr*-KO^m^, treated with LPS. **C** Representative SEM images of murine WT and *Ezr*-KO^m^ primary BM monocytes, cultured on 3D type I collagen hydrogel and untreated or treated with LPS. Scale bar = 5 μm. **D**, **E** Quantification of the volume (**D**) and area (**E**) of primary BM monocytes in both genotypes, of images. Data are represented as mean ± SEM from three independent experiments. Each dot represents an individual cell. Statistical analysis was performed using one-way ANOVA or Tukey’s test for multiple comparisons between the genotype and treatment conditions. **p < 0.01 and ****p < 0.0001. See also Supplementary Fig. S8.

In mouse and human primary MΦs treated with LPS, ezrin localizes in F-actin-rich filopodia [14], cellular structures abundant in adhesion molecules like integrins [41]. In response to LPS, MΦs forms filopodia that act as adhesion sites to the ECM, facilitating MΦ spreading and migration [42–44], and serving as sites for signal transduction [45]. To investigate the effect of ezrin on activation and spreading, we captured different phases of Ly6C^+^ monocyte activation and spreading on a type I collagen (abundant in the lung ECM) surface, in response to LPS over 6 h. Inactivated WT and *Ezr*-KO^m^ monocytes appeared round with small cytoplasm and were enriched in PM F-actin (Fig. 6A^a^, A^e^). Following LPS exposure, WT monocytes showed increased initial lamellipodia formation and ruffling with ezrin/actin co-localization in areas where the PM protruded to form filopodia and retraction fibers (Fig. 6A^b-d^) [46]. However, *Ezr*-KO^m^ monocytes displayed limited lamellipodia and ruffle formation in response to LPS (Fig. 6A^f-h^) and filopodia and retraction fibers were absent. WT Ly6C^+^ monocytes also spread extensively on the collagen surface (area: 111.82 ± 23.75 μm^2^) whereas *Ezr*-KO^m^ Ly6C^+^ monocytes failed to spread (area: 73.60 ± 14.58μm^2^) and had less F-actin (Fig. 6B). Scanning electron microscopy images confirmed the failure of *Ezr*-KO^m^ monocytes to spread in response to LPS (Supplementary Fig. S8A). Similar data were observed when we assessed WT and *Ezr*-KO^m^ bone marrow derived-MΦs (Supplementary Fig. S8B, C).

To gain a higher resolution view of Ly6C^+^ monocytes spreading and F-actin distribution in a microenvironment better resembling the lung ECM, we cultured Ly6C^+^ monocytes in a three-dimensional (3D) collagen-I matrix gel and stimulated with LPS. The collagen hydrogel SEM images revealed increased spreading of the WT monocytes, along with lamellipodia formation, and filipodia/retraction fibers that were directly associated with collagen fibrils in response to LPS. Conversely, the processes were less efficient in *Ezr*-KO^m^ monocytes upon LPS stimulation (Fig. 6C). In the 3D-culture system, we also observed a reduction in cellular volume and spreading in *Ezr*-KO^m^ monocytes compared with WT monocytes in response to LPS (Fig. 6D, E). Confocal images showed that F-actin spreads away from the perinuclear region in WT monocytes in response to LPS (Supplementary Fig. S8D). In contrast, in *Ezr*-KO^m^ monocytes, F-actin remained concentrated around the perinuclear region and quantification showed a 2-fold decrease in PM localized actin intensity (Supplementary Fig. S8D). Altogether, the data show the critical role of ezrin in monocyte interactions with the ECM, involving actin redistribution, lamellipodia formation, filipodia/retraction fiber formation indicative of increased cell adhesion and spreading.

### Ezrin is required for FAK and AKT signaling in monocytes leading to cell adhesion to ECM and survival in response to LPS

**Fig. 7:**
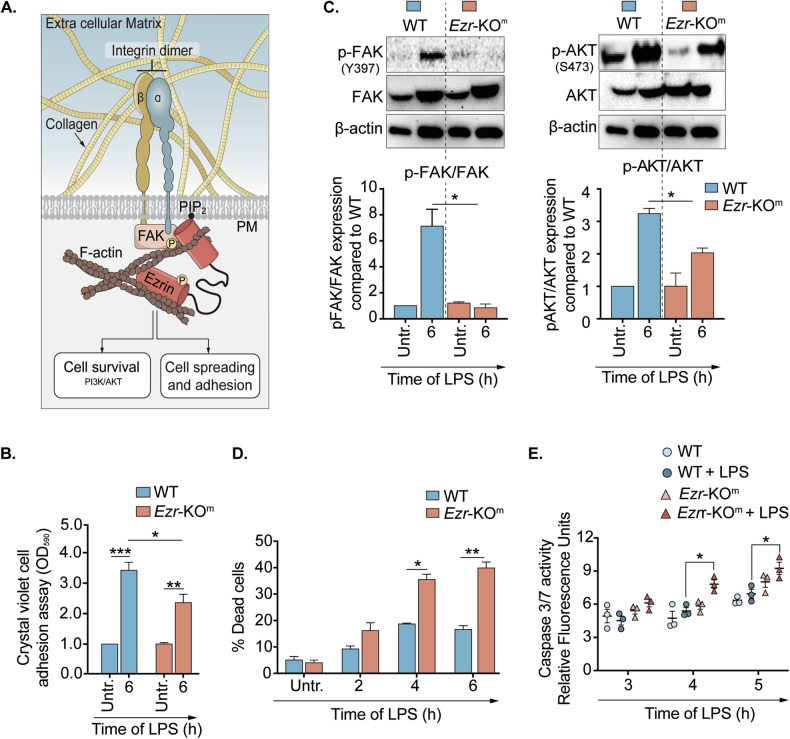
Ezrin is required for FAK and AKT signaling in monocytes leading to cell adhesion to ECM and survival in response to LPS. **A** Schematic representation of ezrin signaling. Ezrin links plasma membrane (PM) and filamentous actin (F-actin) and adheres to the extracellular matrix (e.g., collagen) via integrin α/β. The activation of focal adhesion kinase leads to efficient cell spreading and adhesion, and PI3K/AKT signaling activation is required for cell survival. **B** Quantification of crystal violet absorbance emitted from adherent primary BM monocytes of murine WT and *Ezr*-KO^m^ untreated or treated with LPS (6 h). Absorbance values are relative to WT Untr. **C** Representative WB of phospho-FAK (pFAK, tyrosine 397), total FAK, phospho-AKT (pAKT, serine 473) and total AKT and densitometric analysis of pFAK and pAKT in primary BM monocytes of murine WT and *Ezr*-KO^m^ untreated or treated with LPS (6 h). Bar graphs represent pFAK/FAK and pAKT/AKT ratios. Protein fold increase was normalized to β-actin and relative to untreated cells. **D** Quantification of the percentage of dead cells in primary BM monocytes of murine WT and *Ezr*-KO^m^, post-exposure to LPS. See also Fig. S8C. **E** Relative fluorescence units measuring caspase3/7 activity in primary BM monocytes of murine WT and *Ezr*-KO^m^, untreated or treated with LPS at different time points. Data are represented as mean ± SEM from three independent experiments. Statistical analysis was performed using one-way ANOVA or Tukey’s test for multiple comparisons between the genotype and treatment conditions. *p < 0.05, **p < 0.01 and ****p < 0.001. See also Supplementary Fig. S9.

Based on RNA-seq data, moMs lacking ezrin had altered expression of genes related to focal adhesion and cell-substrate junction assembly pathways in response to LPS (Supplementary Fig. S6C). Notably, it has been previously shown that ezrin interacts with and promotes the activation of FAK (Tyr-397 autophosphorylation) in epithelial LLC-PK1 cells [47]. In immune cells, FAK signaling is downstream of α/β integrin binding to ECM collagen (Fig. 7A) [47–49], creating a docking site for signaling partners (e.g. Src) [47, 50]. This sustains Cdc42, Rac1, and PI3K [45, 51] signaling and F-actin redistribution during activation [52–55]. Therefore, we tested whether ezrin loss impairs the integrin α/β and FAK signaling axis in monocytes responding to LPS, reducing adherence to collagen (Fig. 7A). In line with our hypothesis, we observed that *Ezr*-KO^m^ Ly6C^+^ monocytes adhere less to the type I collagen surface in response to LPS, when compared with WT (Fig. 7B). Although, our RNA-seq data showed a significant decrease in the *Itgα1* (integrin involved in collagen adhesion) expression levels in *Ezr*-KO^m^ moMs and matIMs compared to WT (Supplementary Fig. S9A), no significant differences were observed between genotypes when we assessed the integrin α1 PM levels on moMs and matIMs via flow cytometry (Supplementary Fig. S9B). Additionally, the transcriptomic levels of multiple α and β integrin subunits, which interact with ECM, remained unchanged in response to LPS among moM, tnsIMs and matIMs between WT and *Ezr*-KO^m^ (Supplementary Fig. S9A). However, *Ezr*-KO^m^ Ly6C^+^ monocytes have a profound reduction in FAK phosphorylation compared with WT (Fig. 7C) in response to LPS. These data suggest that loss of ezrin does not affect integrin levels at the PM (although we cannot exclude an effect on integrin clustering [56]) but has a profound effect on the downstream signal transduction associated with monocyte adhesion to ECM.

We further prove the relevance of the FAK signaling on monocyte adhesion and differentiation into MΦs in WT monocytes differentiated to MΦs in vitro with macrophage–colony stimulating factor (mCSF). WT monocytes treated with the FAK inhibitor PND 1186 [57] (Supplementary Fig. S9D) have significantly reduced adhesion to the collagen I surface (Supplementary Fig. S9E) and a high mortality rate (Supplementary Fig. S9F) after 24 h in culture, whereas WT cells treated with vehicle alone adhere and dead cells were not observed. The effect of the drug was specific to monocytes differentiating into MΦs, as no changes in adhesion or survival were observed when the FAK inhibitor was applied to fully differentiated WT MΦs (Supplementary Fig. S9G). Similarly, treatment of *Ezr*-KO^m^ monocytes with a FAK activator Zn27 [58] (Supplementary Fig. S9D) significantly enhanced cell adhesion in response to LPS (Supplementary Fig. S9H) and improved survival (Supplementary Fig. S9I). These findings highlight the critical role of FAK signaling specifically during the early stages of monocyte differentiation into MΦs, which requires adhesion to ECM.

Ezrin also specifically binds to the p85 subunit of PI3K to facilitate activation of the AKT pathway, a key pathway involved in cell survival [14]. Through live imaging studies, we observed that the loss of ezrin resulted in increased blebbing and bursting of monocytes resulting in cell death post 4 h of LPS exposure, up to 6 h (Fig. 7D and Supplementary Fig. S9C; Supplementary Video 1, 2). In line with these phenotypic observations, we observed increased caspase3/7 activity in *Ezr*-KO^m^ Ly6C^+^ monocytes compared with *Ezr*-KO^m^ monocytes in response to LPS, confirming increased cell death in absence of ezrin (Fig. 7E). RNA-Seq data also revealed altered expression of genes such as *CD30* and *Fprl1* regulating cell survival in *Ezr*-KO^m^ MΦs (Fig. 4A, B). Consistent with reduced cell survival and the role of ezrin in facilitating PI3K signaling, we observed a significant reduction in the phosphorylation of AKT in *Ezr*-KO^m^ Ly6C^+^ monocytes compared with WT monocytes (Fig. 7C). Inhibition of AKT with LY294002 (Supplementary Fig. S9D) [33] in WT monocytes was sufficient to reproduce deficiency in cell adhesion (Supplementary Fig. S9E) and cell survival (Supplementary Fig. S9F) on collagen. Conversely, restoring AKT signaling in *Ezr*-KO^m^ monocytes with the AKT activator SC79 (Supplementary Fig. S9D) [59] reduced caspase 3/7 activity (Supplementary Fig. S9I), thereby enhancing the survival of *Ezr*-KO^m^ monocytes compared to those treated with LPS alone. However, AKT activation alone was insufficient to fully restore adhesion (Supplementary Fig. S9H), emphasizing the predominant role of FAK in driving monocyte adhesion. In summary, our data demonstrated that the lack of ezrin in monocytes results in altered cell signaling, leading to defective FAK-mediated adhesion and reduced AKT-dependent cell survival upon activation and differentiation into MΦs.

## Discussion

Macrophage (MΦ) dysfunction plays a significant role in the pathogenesis of several lung diseases including CF, chronic obstructive pulmonary disease (COPD), idiopathic pulmonary fibrosis and asthma [60, 61]. In the context of CF [17, 62], dysfunctional MΦs contribute to non-resolving lung inflammation, impaired bacterial clearance, and promote lung tissue remodeling [19]. Our previous studies unveiled that during MΦ activation, ezrin is induced and phosphorylated downstream of the TLR4 signaling pathway, localizing to the PM and filopodia cell regions [14]. Importantly, our studies also revealed lower levels and impaired PM localization of the ezrin in LPS treated MΦs from pwCF, resulting in reduced activation of anti-inflammatory pathways and compromised host defense against *P. aeruginosa* [14]. These findings prompted us to further explore the role of ezrin in MΦs. In this study, we advance the field by introducing a novel mouse model that enabled us to investigate the in vivo role of ezrin in monocyte/MΦs, specifically in their recruitment to the lungs in response to inflammatory triggers and adaptation to the lung ECM. In response to bacteria and bacterial mediators such as LPS, the lungs are rapidly populated by waves of Ly6C^+^ circulating monocytes. Once within the tissue, moMs gradually acquire tissue-resident MΦs phenotypes based on the specific signals from the lung microenvironment [63]. Interestingly, the absence of ezrin did not impact the emigration of monocytes from the bone marrow to the bloodstream or from the blood into the lung tissues in response to LPS. However, our study asserts the key role of ezrin in monocytes once they populate the lungs, facilitating their adhesion to the lung ECM, which is essential for their survival, proliferation, and differentiation into lung resident IMs. The monocytes/MΦs interaction with the ECM modulates their phenotype [45, 64] by inducing the expression of cell adhesion genes related to integrins, adhesion kinases and MMPs [65, 66]. Our results showed that loss of ezrin in monocytes/MΦs exhibited dramatic redistribution of F-actin and changes in gene expression during LPS activation. This resulted in reduced cellular spreading, reflecting physiological alterations related to ECM adhesion [67, 68] and reduced cell survival in response to LPS. In addition, it identified the impaired activation of FAK and PI3K/AKT signaling as a major driver of these dysfunctions. These findings emphasize the pivotal role of ezrin in monocytes/MΦs adaptation towards the ECM in response to inflammatory stimuli.

Prior studies have highlighted distinct subsets of IMs based on the relative surface expression of multiple markers including *CD11c*, *CD206*, *Lyve-1* and *MHCII* and their localization in lung parenchyma [24, 36]. Our study also revealed the existence of a unique transient population (tnsIMs), which exhibited an intermediate phenotype expressing both Ly6C^+^ and MHCII^+^ markers during the differentiation of moMs to matIMs (Fig. 2B, C). Intriguingly, this subpopulation was detectable exclusively in response to LPS stimulation and was not observed under steady-state conditions (Fig. 2B, C). Our findings align with recent lineage tracing studies that have demonstrated the existence of such a transient population in the lung during the differentiation of classical monocytes to tissue-resident IMs, in a sterile inflammatory model [23]. A similar “transitional” population, expressing both Ly6C^+^ and MHCII^+^ markers, was also observed during the monocyte recruitment into the inflamed colon of mice treated with 2% dextran sodium sulfate [35]. Mechanistically, activation of *Csf1* receptor (*Csf1r*) through its interaction with its ligand *Csf1* is crucial for regulating local MΦ proliferation [69] and maintaining tissue-resident IMs [23]. Our RNA-seq data demonstrated increased *Csf1* expression in tnsIMs compared to moMs and matIMs in LPS-induced WT mice (Fig. 3B, C). Notably, loss of ezrin reduced *Csf1* levels distinctly in tnsIMs (Fig. 3B), as well as proliferation efficiency in LPS-induced tnsIMs and matIMs (Fig. 4B, C), suggested the requirement of ezrin during *Csf1*-mediated cell proliferation and differentiation processes, although the *Csf1r* expression was not affected by loss of ezrin. A scRNA-seq study in mice in which tissue resident IMs were depleted with diphtheria, identified *MafB* as the transcription factor restricting monocyte proliferation and promoting their differentiation toward tissue-resident IMs [23]. In our models in which IM differentiation is driven by LPS, elevated *MafB* levels are observed in moMs compared to tnsIMs and matIMs, suggesting that tnsIMs and matIMs are more prone to acquire a proliferative status in response to infections downregulating *MafB* expression. Nevertheless, the expression of *MafB* is not affected by the loss of ezrin.

During proliferation, cells undergo several rounds of cell division exhibiting cell cycle phases (G0, G1, S, G2 and M) and cell growth. These processes are regulated by specific DNA checkpoints and transcription factors [70]. In a transgenic mouse model of diphtheria-induced lung IM depletion, lung recruited classical monocytes acquired transitional phenotype expressing high levels of S and G2/M cell cycle phase related genes, prior differentiating into IMs. Here, we showed that loss of ezrin disrupts the induction of these cell cycle phases and DNA damage checkpoint genes, specifically in tnsIMs and matIMs, resulting in impaired proliferation. Altogether, impairment in proliferation, in conjunction with cell adhesion deficiency and reduced cell survival drive the loss of tnsIMs number in LPS-treated *Ezr*-KO^m^ mice.

Our study suggested that ezrin has a distinct role in monocytes/MΦs activation as compared with the other ERM family proteins. Indeed, relative to radixin and moesin, ezrin exclusively interacts with specific protein partners [9], such as PI3K-p85 [2, 9] and protein kinase C alpha (PKCα) [71], which is primarily attributed to its conserved NH_2_ terminal domain [6]. Notably, ezrin is also essential for death receptor Fas/CD95-mediated apoptosis [6, 72] and is susceptible to proteolytic degradation by calpain, a calcium-dependent cysteine protease [73, 74]. Moesin is the most abundant ERM expressed protein in monocyte/MΦs, while radixin is expressed at very low levels (Supplementary Fig. S2E and Fig. 3B). Although moesin participates in Toll-like receptor 4 (TLR4) signaling in THP1 cells in response to LPS [75], our data demonstrated that in contrast to ezrin (Fig. 1A-D and Supplementary Fig S2A, E), moesin mRNA and protein levels of in BMD-MΦs, BM-monocytes and primary lung-derived monocytes/ MΦs did not increase in response to LPS and do not compensate for the lack of ezrin (Supplementary Fig. S2A, E). Therefore, the unique molecular interactions and regulatory features of ezrin and its induction in response to activation indicates that its role extends beyond structural support, influencing diverse cellular processes and signaling pathways in response to infections.

In conclusion, this study represents a significant advancement in the field, elucidating the essential role of ezrin in orchestrating the survival, adaptation, and differentiation processes of monocytes as they migrate from the bloodstream to the pulmonary extracellular matrix in response to infectious stimuli. Future studies should investigate the role of ezrin in the context of chronic lung inflammatory models, such as cystic fibrosis and pulmonary fibrosis, since our finding on the profound impact of loss of ezrin on IMs and TGF-β signaling pathway.

### Limitation of the study

The *CX3CR1* promoter is not entirely specific of monocyte/macrophages, as certain cells such as cDCs, eosinophils, and NK cells also express GFP in the *CX3CR1*-GFP mice (see Supplementary Fig. S1B). However, the design of our study specifically enabled us to investigate the impact of ezrin loss within the monocyte/macrophage compartments.

## Materials and methods

### Study design

The aim of this study was to investigate the impact of ezrin on the diversity of lung monocyte/MΦ populations in response to acute inflammation through the analysis of mouse models. Induction of acute lung inflammation was accomplished by administering LPS. The determination of the sample size relied on prior experience and preliminary data. The figure legends specify the number of animals or samples and the repetitions of experiments. All evaluated data points are presented unless otherwise specified.

### Mouse models

A myeloid (monocyte/macrophages)-specific ezrin knocked-out (B6.*Ez*^fl/fl^ mouse [26, 27] crossed with B6.*Cx3cr1* ^tm1.1(cre)Jung/J^; hereafter *Ezr*-KO^m^) mouse model was developed in collaboration with Dr. Gupta from Cleveland clinic. The WT control mice (B6.*Cx3cr1*^tm1.1(cre)Jung/J^) [28, 76], *CCR2* knockout mice (B6.129S4-*Ccr2*^tm1Ifc/J^; hereafter *Ccr2*^−/−^) [77] and *Cx3cr1-*GFP mice (B6.129P2(Cg)-*Cx3cr1*^tm1Litt/J^; hereafter *Cx3cr1*^GFP^) [29] utilized in this study were originally acquired from Jackson Laboratory. All mice were on a C57BL/6 background and bred at the Yale University Animal Facility. Experimental groups were co-housed to control variations due to microbiome effects. For both in vivo and in vitro studies mice aged between 3–5 months were used, ensuring an equal distribution of 50% female and 50% male. All procedures adhered to applicable laws and institutional guidelines and were approved by the Yale University Institutional Animal Care and Use Committee.

### Isolation and culture of murine bone marrow-derived macrophages (BMD-Mφs)

Murine bone marrow cells (BMCs) were collected from 6 to 8-week-old mice using a modified protocol, as described previously [31]. Soft tissue cleared bilateral tibia, femur and pelvic bones were crushed with a mortar and pester to release BM into PBS + 1% FBS + EDTA and the BM suspension was passed through a 100 μm cell strainer and pelleted by centrifugation of 1500 RPM for 5 min. Red blood cells were removed using BD Pharm Lyse^TM^ RBC lysing buffer (BD Bioscience, NJ). BMCs were resuspended in Dulbecco’s Modified Eagle’s high glucose medium (DMEM; Corning, NY) media (DMEM supplemented with 10% FBS, 1% l-glutamine and 1% pen-strep). After overnight culture, at 37 °C with 5% CO_2_, non-adherent BMCs were differentiated to BMD-Mφs for 7 days in 20 ng/ml recombinant murine-CSF (ConnStem Inc., CT, USA). After 7 days, cells were detached using trypsin and characterized by flow cytometry (F4/80^+^/MAC-1^+^ population). The day before the experiments, cells were plated at the density of 1 × 10^6^ cells/well (6 well plates) and treated with PA-LPS (1 μg/mL; L9143 Millipore Sigma) for 6 h and 24 h with continued incubation at 37 °C with 5% CO_2_. In a set of experiments, BMD-Mφs were plated at the density of 200,000 cells/well (96 well plates) and treated with FAK inhibitor (PND 1186—1 μM, Tocris) or AKT inhibitor (LY 294002—10 μM, Tocris) for 24 h with continued incubation at 37 °C with 5% CO_2_.

### Isolation and treatment of murine bone marrow Ly6C^+^ monocytes (BM-monocytes)

BM cells were isolated from mouse bone marrow as described in the previous section. Ly6C^+^ monocytes were isolated using mouse monocyte isolation kit (BM), (Miltenyi Biotec) as per manufacturer’s instructions. Monocytes were cultured on type I collagen-coated 12-mm round coverslips at 70–80% confluence in DMEM media containing 5 ng/ml recombinant murine-CSF (ConnStem Inc., CT, USA). After 1 h of incubation at 37 °C with 5% CO_2_, cells were stimulated with FAK inhibitor (PND 1186—1 μM, Tocris) or AKT inhibitor (LY 294002—10 μM, Tocris) or FAK activator (Zn27—100 nM, MedChem Express) or AKT activator (Sc79—2 μM, MedChem Express) and/or with PA-LPS (1 μg/mL; L9143 Millipore Sigma) for up to 6 h or 24 h with continued incubation at 37 °C with 5% CO_2_.

### Acute LPS model and in vivo studies

The acute LPS model was adapted from our previous work [78]. Mice were nebulized with 12.5 mg of *Pseudomonas aeruginosa* (PA)-LPS dissolved in 5 ml of PBS (L9143 Millipore Sigma) for 15 min using a nebulizer (Pulmo-Aide Compressor; Natallergy, Duluth, GA). 24 h post-nebulization, mice were euthanized via intraperitoneal administration of 8% urethane in PBS. Blood, bronchoalveolar lavage fluid (BALF) and lung tissues were then collected post 24 h [19, 79]. For the in vivo proliferation studies, mice received intraperitoneal injection with BrdU (4 mg/mL) 24 h before LPS nebulization. To maintain the BrdU levels, BrdU power (0.8 mg/mL; B5002; Sigma-Aldrich) was administrated in combination with sucrose (0.5%; S0389; Sigma-Aldrich) in the drinking water throughout the experiment.

### Blood, bronchoalveolar lavage fluid (BALF) and lung tissue collection

#### Blood

Mice were anesthetized and up to 500 μl of blood was recovered by retro-orbital puncture (terminal procedure). 50 μl of blood was used to analyze complete blood counts (CBCs) by Hemavet 950 (Drew Scientific). 150 μl of blood was treated with 1 mL of RBC lysis buffer (eBio-science) for 45 min, then centrifuged at 2800RPM for 7 min. Cell pellet was resuspended in 1 mL RBC lysis buffer for 15 min, then centrifuged at 2800 RPM for 7 min and cleared cell suspension was collected in 400 μl of PBS and used for flow cytometry.

#### BALF

BALF was collected using standard methods with 2 mL BAL solution (PBS with 5 mM EDTA and cOmplete Mini EDTA-free protease inhibitor cocktail, Roche; 1 tablet per 25 mL). BALF was centrifuged at 1500 rpm for 5 min and supernatants were collected and stored at −80 °C until analysis. Cell pellets were resuspended in RBC lysis buffer for 1 min, centrifuged at 1500 rpm for 5 min and cell suspension was collected in 200 μl of PBS, counted, and used for flow cytometry for differential cell counting

#### Lung tissues

For lung collection, mice underwent a midline incision from sternum to diaphragm, and blood was removed from the pulmonary circulation through transcardial perfusion of PBS supplemented with heparin (1:1000). The right lobes were tied off and the left lung lobes were inflated via intratracheal catheter with 0.5% UltraPure Low Melting Point agarose (Invitrogen) in PBS at a constant pressure. Subsequentially, left lobes were harvested, fixed overnight in 10% neutral buffered formalin, embedded in paraffin in longitudinal orientation and used for immunohistochemistry. The right inferior lobes were collected in PBS for flow cytometry analysis and RNA later solution for mRNA expression.

### Immunohistochemistry

The mouse lung tissue immunohistochemistry procedure was performed as described previously. Mouse lung tissue’s left lobe was inflated with 0.5% Agarose solution and subsequently fixed with formalin overnight. Fixed tissues were paraffin-embedded and sectioned by Yale Pathology Tissue Services following standard procedures. Formalin-fixed and paraffin-embedded sections were deparaffinized with xylene and gradually rehydrated using a series of graded ethanol solutions. Sections were then washed with deionized water. After antigen retrieval and blocking, sections were incubated with rat monoclonal anti-CD68 antibody (1:100, Bio-Rad) at 4 °C overnight. Sections were washed in PBS and then incubated with a fluorescent-labeled anti-rat (Alexa 564) antibody at a dilution of 1:400 for 2 h at room temperature. DAPI was used for nuclei counterstaining. Slides were then washed with PBS and mounted with Prolong Gold (Invitrogen). Images were captured using a Leica THUNDER Imager Live cell and 3D Microscope. CD68 positive cells across whole lung specimens were counted using ImageJ. Upon setting background fluorescence as threshold, cells expressing intensity above threshold were counted as CD68 positive cells. Data are represented as mean ± SEM from three biological replicates per genotype.

### Immunofluorescence

BMD-Mφs and Ly6C^+^ monocytes with or without LPS treatment were fixed using 4% paraformaldehyde for 15 min at room temperature. Cells were blocked and permeabilized for 60 min in PBS/20% donkey serum/0.1% saponin, and stained, as indicated. Rabbit monoclonal anti-phospho-ezrin (1:100, SAB) and rabbit polyclonal anti-Ezrin (1:250, Abcam) antibodies were used; directly conjugated fluorescent secondary antibody (ThermoFisher) was used at 1:300 dilutions, 1 h at room temperature. Cells were washed with PBS and incubated with Phalloidin (1 drop/mL) for 30 mins at room temperature. Cells were washed with PBS and incubated with DAPI 1:1000 for 5 min and mounted with Prolong Gold. Images were captured with a Leica THUNDER Imager Live cell and 3D Microscope using a 63× objective lens. For each experiment, at least six different fields were acquired.

### FACS and flow cytometry staining

Lung inferior lobes (or whole lungs for sorting) were processed to prepare single-cell suspensions using C tubes, a lung dissociation kit (Miltenyi, CA), and a GentleMACS dissociator (Miltenyi, CA) as per the manufacturer’s instructions. Homogenized lung tissues were then filtered through a 100 μm cell strainer to achieve a single-cell suspension followed by RBC lysis using BD Pharm Lyse (BD Biosciences, CA). After cell counting, 1 × 10^6^ cells were used for staining, while up to 1 × 10^7^ whole lung cells were used for sorting. Cells were initially stained with a LIVE/DEAD Fixable Aqua Dead Cell Stain Kit (ThermoFisher Scientific), then incubated with FcBlock (BD Biosciences, CA), and subsequently stained with freshly prepared antibody cocktails for 30 min at 4 °C. For cell sorting, cells were washed and sorted using a FACSAria II (BD Biosciences). For flow cytometry, cells were washed and fixed with BD Cytofix/Cytoperm, (BD Biosciences, CA) and analyzed on a BD LSR II instrument using BD FACSDiva software. Data analysis was performed using FlowJo software (TreeStar, Ashland, OR). Total live cell counts were determined by multiplying the percentage of each assessed population among singlet/live cells by the total lung cell counts in the inferior lobes.

### Flow cytometry gating strategy

The gating strategy was adapted from Oz, H.H. et al 2022 [19] and is depicted in Supplementary Fig. S1D. Singlet events were gated followed by exclusion of dead cells stained with LIVE/DEAD Fixable Aqua Dead Cell Stain Kit (ThermoFisher Scientific). Debris was excluded by the exclusion of FSC-H SSC-H small events, and subsequent gating was applied to isolate CD45^+^ cells. CD45^+^ cells were further gated for CD11c^+^ and CD11b^+^ cells. Alveolar macrophages (AMs) were distinguished by selecting CD11c^+^ cells. Within this population, dendritic cells (DCs) were discerned from AMs by their expression levels of CD64/CD24, with DCs identified as CD24^high^ CD64^neg^. From the CD11c^−^ fraction, myeloid cells were gated based on CD11b expression. Granulocytes were further delineated from CD11b^+^ cells by their expression of CD24 and MHC-II, with subsequent subdivision into neutrophils (Ly6G^+^ SiglecF^−^) and eosinophils (Ly6G^−^ SiglecF^+^). The non-granulocytic fraction was segregated into Ly6C^−^ moMs, moMs (Ly6C^+^ MHC-II^−^), tnsIMs (Ly6C^+^ MHC-II^+^) and matIMs (Ly6C^+^ MHC-II^-^).

### RNA sequencing and data analysis

FACS-sorted moMs, tnsIMs and matIMs were subjected to extract the RNA using miRNEasy Micro kit (Qiagen, MD). The RNA integrity (RIN) and quantity was determined on the Agilent 2100 Bioanalyzer (Agilent, CA) with Agilent RNA 6000 Pico Kit (Agilent, CA). Samples with RIN ≥ 8.0 were used for library preparation. Reverse transcription and cDNA preamplification were performed using the SMART-Seq v4 Ultra Low Input RNA Kit for Sequencing (Takara Bio USA, MI), and sequencing libraries were prepared using Nextera XT DNA Sample Preparation kit (Illumina, CA, USA), according to manufacturers’ instructions. Libraries were pooled and sequenced on Illumina NovaSeq 6000 (Illumina, CA,) using 150 paired-end sequencing. After quality control (FastQC), reads generated from each sample were aligned to the mouse genome (GRCm39) with STAR (version 2.7.9a, –quantMode GeneCounts), using the Gencode M29 transcript annotation as transcriptome guide. Normalization with the TMM method and identification of differentially expressed genes were performed with the edgeR package in Bioconductor (https://bioconductor.org). Differentially expressed genes were identified with the glmQLFTest function using a double threshold on gene expression changes and associated statistical significance (absolute log2 fold change > 0.75, *p*-value < 0.05). Functional annotation and enrichment analysis were performed with the enrichR package (https://maayanlab.cloud/Enrichr) and MetaCore (https://portal.genego.com/). Sequencing data was generated from 3 samples per genotype.

### Cell morphology using 3D collagen hydrogels

3 mg/ml collagen hydrogels were polymerized for 1 h at 37 °C from a 9.48 mg/ml stock solution (Corning) onto dopamine hydrochloride (Sigma) coated plates. During the polymerization process, cells were seeded, and the gels were flipped frequently to achieve 3D embedding of cells. Subsequently, treatment was initiated, and at indicated time points, hydrogels were fixed with 4% paraformaldehyde (Santa Cruz). Samples were washed three times with PBS (Invitrogen), permeabilized with 0.3% Triton-X, and blocked with 2% BSA for 2 h at room temperature. Cells were stained with phalloidin (Invitrogen) for 2 h, washed and counterstained with Hoechst. For 3D analysis, confocal image stacks were taken on Leica SP8 laser scanning confocal microscope and deconvolved using Huygens (Scientific Volume Imaging). Volumes were assessed using Imaris (Oxford Instruments) where the cytoplasm was identified using actin and the nuclei segmented using Hoechst. A macro was designed with a set threshold for each channel and was run for all the images for the two time points.

As a secondary analysis, collagen sheets were fabricated between polydopamine treated coverslips and BSA treated coverslips, where after polymerization, the BSA coverslips were removed using PBS. After washing three times with PBS, cells were seeded onto the collagen sheets and allowed to attach for 10 min. Treatments were initiated and samples were fixed at the indicated time points using 4% paraformaldehyde. Samples were washed before dehydrating using a series of ethanol and hexamethyldisilazane solutions, coated with iridium, and imaged on a SU-70 scanning electron microscope (Hitachi). Cell morphology was assessed using ImageJ for 2D SEM image analysis where significance was determined by two-way ANOVA using a Tukey post hoc test.

### Scanning electron microscopy

Cells were fixed with 4% paraformaldehyde (Electron Microscopy Sciences) in 0.1 M phosphate buffer for 20 min at 4 °C and 2.5% glutaraldehyde (Electron Microscopy Sciences) in 0.1 M phosphate buffer for 1 h at room temperature. The samples were washed three times for 5 min each in 0.1 M phosphate buffer, postfixed with 1% osmium tetroxide (Electron Microscopy Sciences) in 0.1 M phosphate buffer for 45 min, and washed three times for 7 min each in ice-cold distilled water. Cells were dehydrated with a graded series of ethanol concentrations (50, 70, 85, 95, and 100% ×3 for 7 min each) and then were critical-point dried with a Tousimis Autosamdri 810 critical point dryer (Tousimis). Samples were then coated with 2 nm of platinum (Pelco) with a high-energy ion-beam sputtering system (Bal-Tec SCD 005 sputter coaters). Cells were imaged with a Zeiss Crossbeam 550 FIB-scanning electron microscope (https://medicine.yale.edu/ccmi/em/).

### In-vivo proliferation assay

The BrdU and Ki67 proliferation assay was performed as previously described in Hogan et al. [80]. WT and *Ezr*-KO^m^ mice were administered BrdU (4 mg/mouse) (Catalogue # 55961, BD Biosciences) via intraperitoneal injection, 24 h before LPS nebulization. BrdU concentration was maintained in their drinking water (0.8 mg/mL in combination with % 0.5 sucrose). LPS nebulization and preparation of single-cell suspensions from lung inferior lobes, followed by extracellular FACS staining were performed as described above. Further, cells were intracellularly co-stained with anti-BrdU conjugated with FITC (1:50) (Catalogue # 55961, BD Biosciences) and anti-Ki67 conjugated with PE (1:200) (Catalogue # 50-5698, Thermo Fisher Scientific, eBioscience^TM^) antibodies [80]. Subsequently, BrdU^+^ and Ki67^+^ populations were analyzed using flow cytometry.

### Cell death assay

The rate of cell death between LPS-stimulated WT and *Ezr*-KO^m^ *Ly6C*^*+*^ monocytes was quantified by measuring caspase 3/7 activity. 96-well microplates with black walls and a clear bottom were coated with type-I collagen at 37 °C for overnight. After coating, the plates were washed with washing buffer (0.1% BSA in DMEM media) and blocked with 0.5% BSA in DMEM media at 37 °C for 1 h in a CO_2_ incubator. 200,000 cells per well were plated in triplicates in DMEM media with recombinant m-CSF (5 ng/mL or 20 ng/mL; Conn Stem). Subsequently, cells were treated with PND 1186 (1 μM, Tocris) or LY 294002 (20 μM, Tocris) or Zn27 (10 nM, Medchem Express) or Sc79 (2 μM, Medchem Express) and/or with PA-LPS (1 μg/mL; L9143 Millipore Sigma) for up to 6 h or 24 h with continued incubation at 37 °C with 5% CO_2_. During this incubation period, caspase 3/7 activity was detected using Immunochemistry Technologies (ICT) Magic Red® Caspase-3/7 Assay Kit, according to the manufacturer’s instructions (Catalog #936; Dusseldorf, Germany). Fluorescence intensity was measured using a Molecular Devices Gemini XS fluorometric plate reader set at 590 nm excitation, 640 nm, emission, with a 630 nm cut-off filter.

### Cell adhesion assay

96 well plates were coated with type-I collagen as mentioned in the previous method. 100,000 cells per well were plated in triplicates in DMEM media with recombinant m-CSF (5 ng/mL or 20 ng/mL; Conn Stem). Subsequently, cells were treated with PND 1186 (1 μM, Tocris) or LY 294002 (20 μM, Tocris) or Zn27 (10 nM, Medchem Express) or Sc79 (2 μM, Medchem Express) and/or with PA-LPS (1 μg/mL; L9143 Millipore Sigma) for 6 h or 24 h with continued incubation at 37 °C with 5% CO_2_. After incubation, the plates were shaken at 2000 rpm for 10–15 seconds and washed 2–3 times with washing buffer. The cells were fixed with 4% paraformaldehyde and incubated at room temperature for 10–15 min before another wash with washing buffer. Next, the plates were stained with Crystal Violet (5 mg/ml in 2% ethanol) for 10 min, followed by washing with water. Once dried, 50 μl of 1% SDS (in water) was added to each well and the plates were incubated at room temperature for 30 min and recorded the absorbance at 550 nm (OD_550_).

### Live cell imaging

Time-lapse imaging was performed on LPS treated/untreated Ly6C^+^ monocytes using Leica THUNDER Imager Live cell and 3D Microscope with brightfield and FITC channels (*λ*_ex/em_ = 488/520). Cells were imaged every one hour, with continued incubation of cells at 37 °C for up to 6 h. The exposure time was set to 30 ms for the FITC channel. The imaging fields were chosen randomly at multiple spaces and monitored them throughout the imaging. Morphological changes were identified and quantified the counts using ImageJ. Based on the morphological changes such as membrane blebbing/cell disruption and spillover of DAPI/FITC intensity throughout the cell, the cells were masked and counted referring to as dead cells at each time point.

### Quantitative RT-PCR analysis

LPS treated/untreated BMD-Mφs, Ly6C^+^ monocytes, lung-sorted cells and lung tissues were lysed in TRIzol and the total RNA was isolated using an RNeasy Mini Kit (Catalogue #74104; Qiagen). DNAse contamination was removed using an RNAse‐free DNA Set (Catalogue #79254; Qiagen) by following the manufacturer’s instructions. RNA concentration was measured by Nanodrop. cDNA was synthesized using *SuperScript*™ *III Reverse Transcriptase* kit (Catalogue #18080-093; Invitrogen) as per the manufacturers’ instructions. Real-time PCR analysis was performed with a Bio-Rad iCycler using TaqMan technology with primers and probes purchased from Applied Biosystems (Life Technology). The levels of respective genes relative to the Stx5a rRNA were analyzed, and relative expression or fold change compared to control was calculated by the ∆∆C_T_ method.

### Bradford protein assay

BALF supernatant total protein concentrations were determined using the Bradford assay. Bovine serum albumin (BSA) was used as the standard, with a range of 0–5 mg/mL prepared in phosphate-buffered saline (PBS). LPS-treated/untreated WT and *Ezr*-KO^m^ mice BALF supernatant samples were appropriately diluted with PBS in a 1:5 ratio. For each standard and sample, 10 µL was pipetted into a 96-well microplate, followed by the addition of 200 µL of Bradford reagent. After gentle mixing, the plate was incubated at room temperature for 5 min. Absorbance was measured at 595 nm using a microplate reader. A standard curve was generated from the BSA standards, and protein concentrations were interpolated from the curve based on sample absorbance. All measurements were performed in triplicate.

### Western blot (WB)

Cells were lysed using RIPA lysis buffer (Cell Signaling) containing 1 mM phenylmethane sulfonyl fluoride (PMSF), protease and phosphatase inhibitor cocktails (Roche Diagnostics) with incubation on ice for 30 min. Further, lysates were centrifuged at 12,000*g* for 15 min, recovered supernatant and quantified total protein concentration using Bradford assay (BioRad Laboratories, CA), following manufacturer’s protocol. An equal amount of protein was separated by SDS-PAGE on 4–15% Mini PROTEAN Gels (Bio-Rad Laboratories, CA), transferred to PVDF membrane (Bio-Rad Laboratories, CA) and incubated with primary antibodies overnight at 4 °C. HRP conjugated to IgG secondary antibodies (1:2000; Santa Cruz Biotechnology) and Clarity ECL western blotting substrate (Bio-Rad Laboratories, CA) were used for detection. The following primary antibodies were used: rabbit mAb anti-pezrin (1:1000, SAB), rabbit polyclonal anti-ezrin (1:1000, Abcam), rabbit polyclonal anti-moesin (1:1000, Cell signaling technology), rabbit polyclonal anti-pFAK and anti-FAK (1:1000, Cell signaling technology), rabbit polyclonal anti-pAKT and anti-AKT (1:1000, Cell signaling technology) and rabbit-HRP anti-actin (1:5000, Santa Cruz). The chemiluminescence imaging system ChemiDoc (BioRad) and the Image lab software (BioRad) were used for image acquisition. Blot signal intensities were quantified using FIJI (ImageJ). Densitometric analysis was performed by normalizing the respective bands to loading control (β-actin).

### Quantification and statistical analysis

The data, represented as mean ± SEM, were visualized using GraphPad Prism software (GraphPad, CA, USA). Statistical significance was determined using one-way ANOVA and Tukey’s test for multiple comparisons or Student’s t-test, with a significance threshold set at *p < 0.05, **p < 0.01, ***p < 0.001 and ****p < 0.0001. In vitro experiments for Figs. 1, 6 and 7 were conducted independently at least three times. In vivo experiments for Fig. 2 were conducted independently at least three times with three or more samples per group each. The datasets used in Fig. 5 are derived from one experiment with six mice per group. The dataset used in Supplementary Fig. S5D data represents technical replicates of lung-sorted cells pooled from three mice per group.

## Supplementary information

Supplementary Information

Uncropped western blots

Dataset Fig. 1 to 7 and Supplementary Fig. S1 to S9

Video 1

Video 2

## Data Availability

The bulk RNA-seq files are available in GEO (GSE267506) database. All data needed to obtain the conclusions in this work are present in the paper or the Supplementary Materials figures or tables. All mouse lines, reagents, and software used are listed in the paper or the Supplementary Materials. Any other information will be available upon request.

## Inclusion and diversity

We support inclusive, diverse, and equitable conduct of research.
