# Supplementary material for: Ezrin drives adaptation of monocytes to the inflamed lung microenvironment
Source: Cell Death Dis. 2024 Nov 29;15(11):864. doi: 10.1038/s41419-024-07255-8 (PMC11607083; doi:10.1038/s41419-024-07255-8)
Supplement: Supplementary file 1 — Supplementary Information [file 41419_2024_7255_MOESM1_ESM.docx]

**SUPPLEMENTARY INFORMATION**

**Ezrin drives adaptation of monocytes to the inflamed lung microenvironment.**

Ravindra Gudneppanavar^1#^, Caterina Di Pietro^1#^, Hasan H Öz^1^, Ping-Xia Zhang^1,2,3^, Ee-Chun Cheng^1^, Pamela H Huang^1^, Toma Tebaldi^2,4,5^, Giulia Biancon^2,4^, Stephanie Halene^2,4^, Adam D Hoppe^6^, Catherine Kim^7^, Anjelica L Gonzalez^7^, Diane S Krause^2,3,8^, Marie E Egan^1,9^, Neetu Gupta^10^, Thomas S Murray^1^, Emanuela M Bruscia^1,2*^

**AFFILIATIONS**

*^1^Department of Pediatrics, School of Medicine, Yale University, New Haven, CT, USA*

*^2^Yale Stem Cell Center, School of Medicine, Yale University, New Haven, CT*

*^3^Department of Laboratory Medicine, School of Medicine, Yale University, New Haven, CT, USA*

*^4^Department of Hematology, School of Medicine, Yale University, New Haven, CT, USA*

*^5^Department of Cellular, Computational and Integrative Biology (CIBIO), University of Trento, Trento, Italy*

*^6^Department of Chemistry and Biochemistry, South Dakota State University, Brookings, SD, USA*

*^7^Department of Biomedical Engineering, Yale University, New Haven, CT, USA*

*^8^Department of Pathology, School of Medicine, Yale University, New Haven, CT, USA*

*^9^Department of Cellular and Molecular Physiology, Yale University, New Haven, CT, USA*

*^10^Department of Inflammation and Immunity, Cleveland Clinic Foundation, Cleveland, OH, USA*

^#^ These authors contributed equally to this work

***Corresponding author.** Email: [emanuela.bruscia@yale.edu](mailto:emanuela.bruscia@yale.edu) Phone: (203) 737-5556

**Conflict of interest:** The authors declare no conflict of interest.

**SUPPLEMENTARY FIGURES:**

**Supplementary Fig. S1.** Validation of the monocyte/macrophages (MΦs)-specific ezrin knock-out (*Ezr*-KO^m^) mouse model.

**Supplementary Fig. S2.** Radixin and Moesin expression remain unaltered in monocytes and MΦs in response to LPS.

**Supplementary Fig. S3.** Loss of ezrin exhibits a decreased number of CD68^+^ MΦs in *Ezr*-KO^m^ lung tissue in response to LPS.

**Supplementary Fig. S4.** Characterization of lung and blood immune cells in WT and *Ezr*-KO^m^ mice at steady state and in response to LPS.

**Supplementary Fig. S5.** *Ezr*-KO^m^ mouse have a temporary increased lung inflammation in response to LPS compared to controls.

**Supplementary Fig. S6.** Characterization of lung macrophage gene expressions, differentially expressed genes (DEGs) and enrichment pathways in WT and *Ezr*-KO^m^ mice in response to LPS.

**Supplementary Fig. S7.** Monocyte/MΦs lacking ezrin exhibit proliferation defects in response to LPS.

**Supplementary Fig. S8.** Ezrin is required for efficient monocyte/MΦ filipodia formation and cell spreading during activation with LPS.

**Supplementary Fig. S9.** Loss of ezrin does not alter monocytes/MΦ integrin expressions but alters downstream FAK/AKT signaling pathways in Ly6C^+^ monocytes in response to LPS.

**
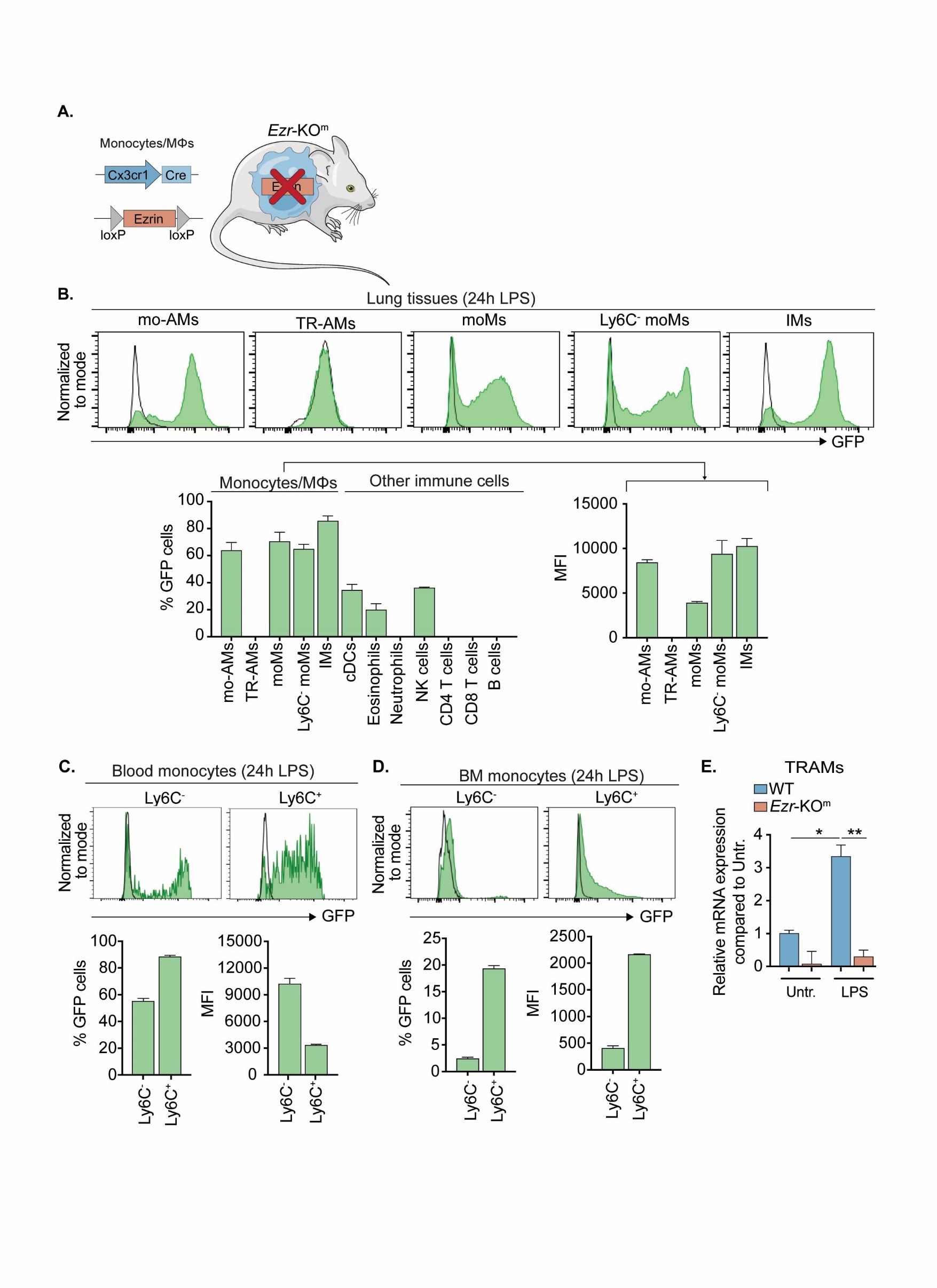
**

**Supplementary Fig. S1**

**Supplementary Fig. S1. Validation of the monocyte/macrophages (MΦs)-specific ezrin knock-out (*Ezr*-KO^m^) mouse model.**

(A) Cartoon representation of mouse model with ezrin knocked out (KO) in monocytes/MΦs (*Ezr*-KO^m^). The mouse model was obtained by crossing ezrin flox/flox mouse (B6.*Ez*^fl/fl^) with *Cx3cr1* Cre^+^ (B6.*Cx3cr1*^tm1.1(cre)Jung/J^) mouse. (B) Histograms showing the fluorescence intensity of *Cx3cr1*^GFP^ (B6.129P2(Cg)-*Cx3cr1*^tm1Litt/J^) staining in the lung MΦs such as monocyte derived alveolar MΦs (mo-AMs), tissue resident alveolar MΦs (TR-AMs), Ly6C^+^ monocyte derived MΦs (moMs), Ly6C^-^ moMs, interstitial macrophages (IMs). Bar graphs depicting the flow cytometry analysis of the percentage of GFP-positive cells among different monocyte-derived MΦs and other immune cells (bottom left) and their corresponding mean fluorescence intensities (MFI) (bottom right). (C-D) Flow analysis of GFP-positive cells in LPS-induced *Cx3cr1*^GFP^ reporter mouse model depicting as histograms (top), their percentages (bottom left) and MFIs (bottom right) in both blood (C) and bone marrow (BM) (D) Ly6C^+^ and Ly6C^-^ monocytes. (E) Quantitative PCR (qPCR) of ezrin in murine WT and *Ezr*-KO^m^ TRAMs, untreated or treated with LPS. The relative mRNA expression of ezrin was normalized to STX5a and the WT untreated (Untr.). Data were generated from three independent experiments. Histogramas are depicted as normalized to mode and the bar graphs are depicted as means ± SEM. Statistical significance was tested by One-way ANOVA and Tukey’s multiple comparisons test between genotypes for each time point separately (* p<0.05; ** p<0.01). Related to Fig. 1.

**
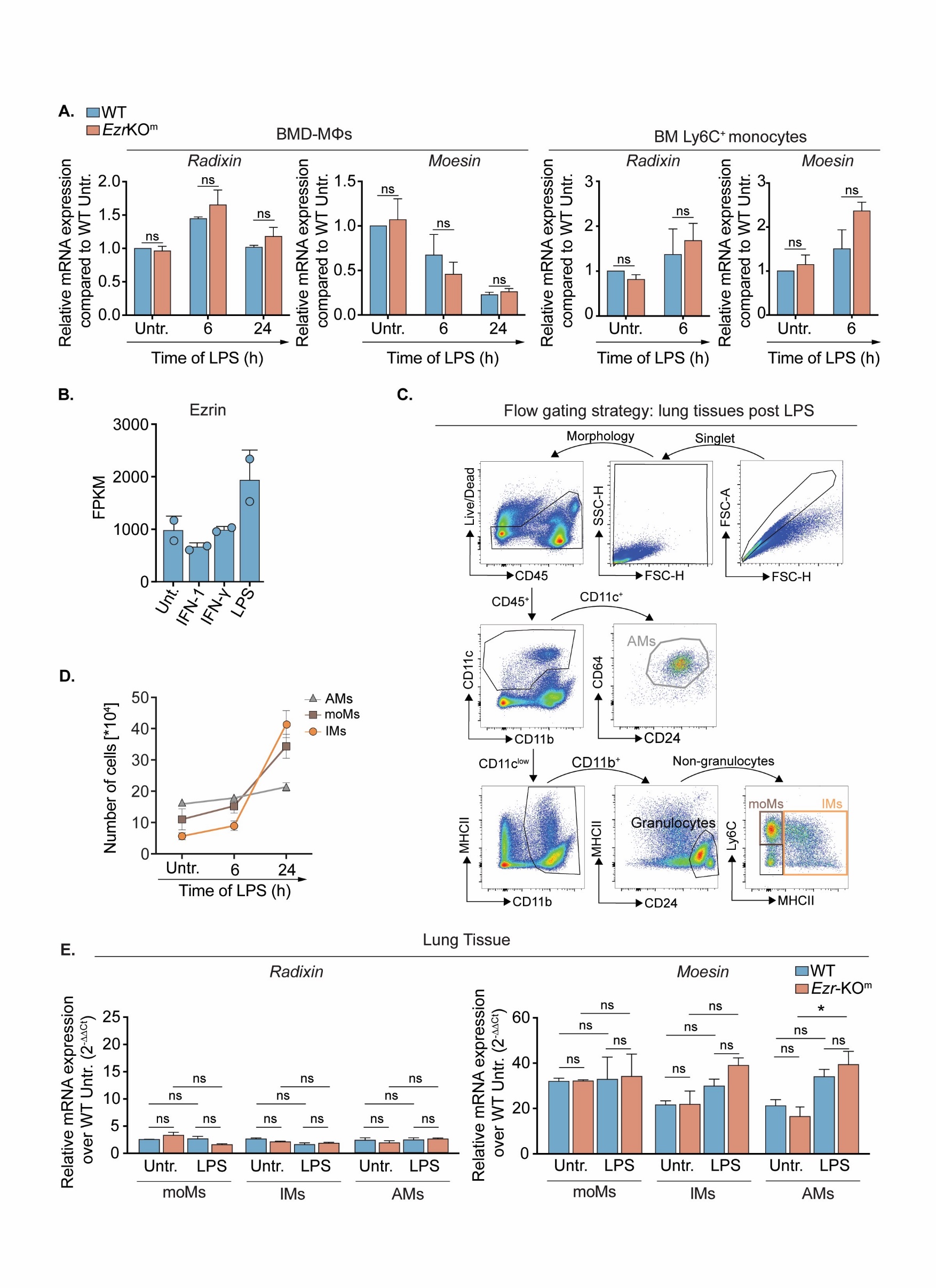
**

**Supplementary Fig. S2**

**Supplementary Fig. S2. Radixin and Moesin expression remain unaltered in monocytes and MΦs in response to LPS.**

(A) Quantitative PCR (qPCR) for radixin and moesin in WT and *Ezr*-KO^m^ mouse bone marrow-derived macrophages (BMD-MΦs) (A) and primary bone marrow (BM) Ly6C^+^ monocytes (B), untreated or treated with LPS for 6h and/or 24h. The relative mRNA expressions of radixin and moesin were normalized to STX5a and the WT untreated (Untr.). (B) Bulk RNA-Seq analyzed transcriptional expressions of ezrin in WT BMD- MΦs treated with interferon alpha1, interferon gamma and LPS. (C) Flow cytometry gating strategy on lung inferior lobe single cell suspension for assessing immune cells. The strategy was adapted from H. H. Oez et al 2022., see STAR methods for a detailed description. (D) Quantification of immune cell numbers (monocyte-derived MΦs (moMs), interstitial MΦs (IMs) and alveolar MΦs (AMs)) by fluorescence activated cell sorting (FACS) in the inferior lobe of LPS-induced WT mice in a time-dependent manner. (E) qPCR of radixin and moesin in murine WT and *Ezr*-KO^m^ moMs, IMs and AMs, untreated or treated with LPS. The relative radixin and moesin mRNA expression was normalized to STX5a and the WT Untr., distinctly for each phenotype. Data were generated from three independent experiments. Bars are depicted as means ± SEM, significance was tested by One-way ANOVA and Tukey’s multiple comparisons test between genotypes for each time point separately (ns – non significant). Related to Fig. 1.

**
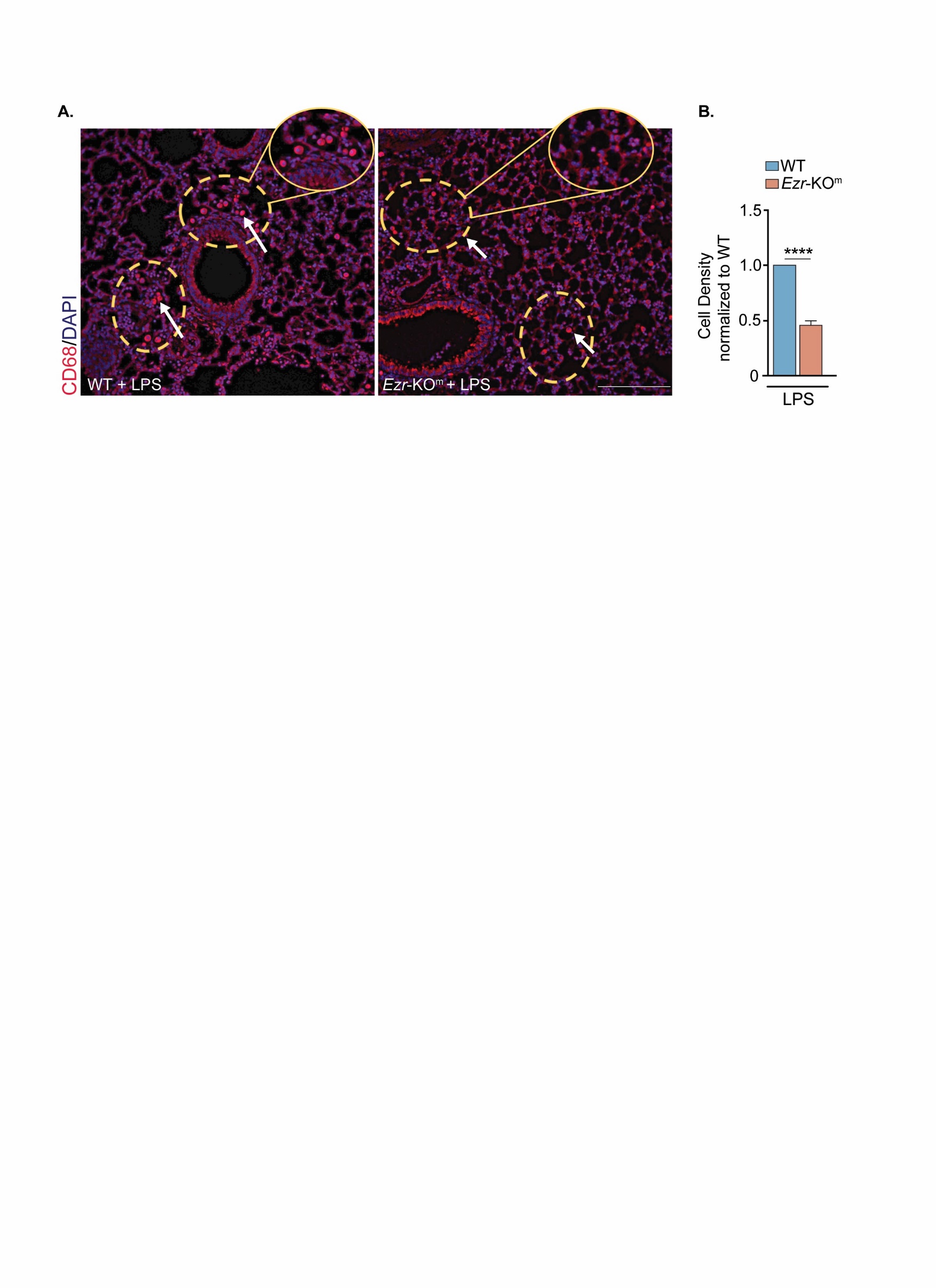
**

**Supplementary Fig. S3**

**Supplementary Fig. S3. Loss of ezrin exhibits a decreased number of CD68^+^ MΦs in *Ezr*-KO^m^ lung tissue in response to LPS.**

(A) Representative immunofluorescence staining for CD68 (red) and DAPI (blue) in lung tissues of LPS-treated WT and *Ezr*-KO^m^ mice. Scale bar = 166.7 μm. (B) Quantification of CD68^+^ events in lung tissues of WT and *Ezr*-KO^m^ mice. Events were counted from 10 randomly selected 1200 x 1200 pixel^2^ windows on the lung tissues of 3 mice. The bar graph depicts CD68^+^ cell density normalized to WT. Bars are depicted as means ± SEM, significance was tested by student’s t-test between genotypes (****p < 0.0001). Related to Fig. 2.

**
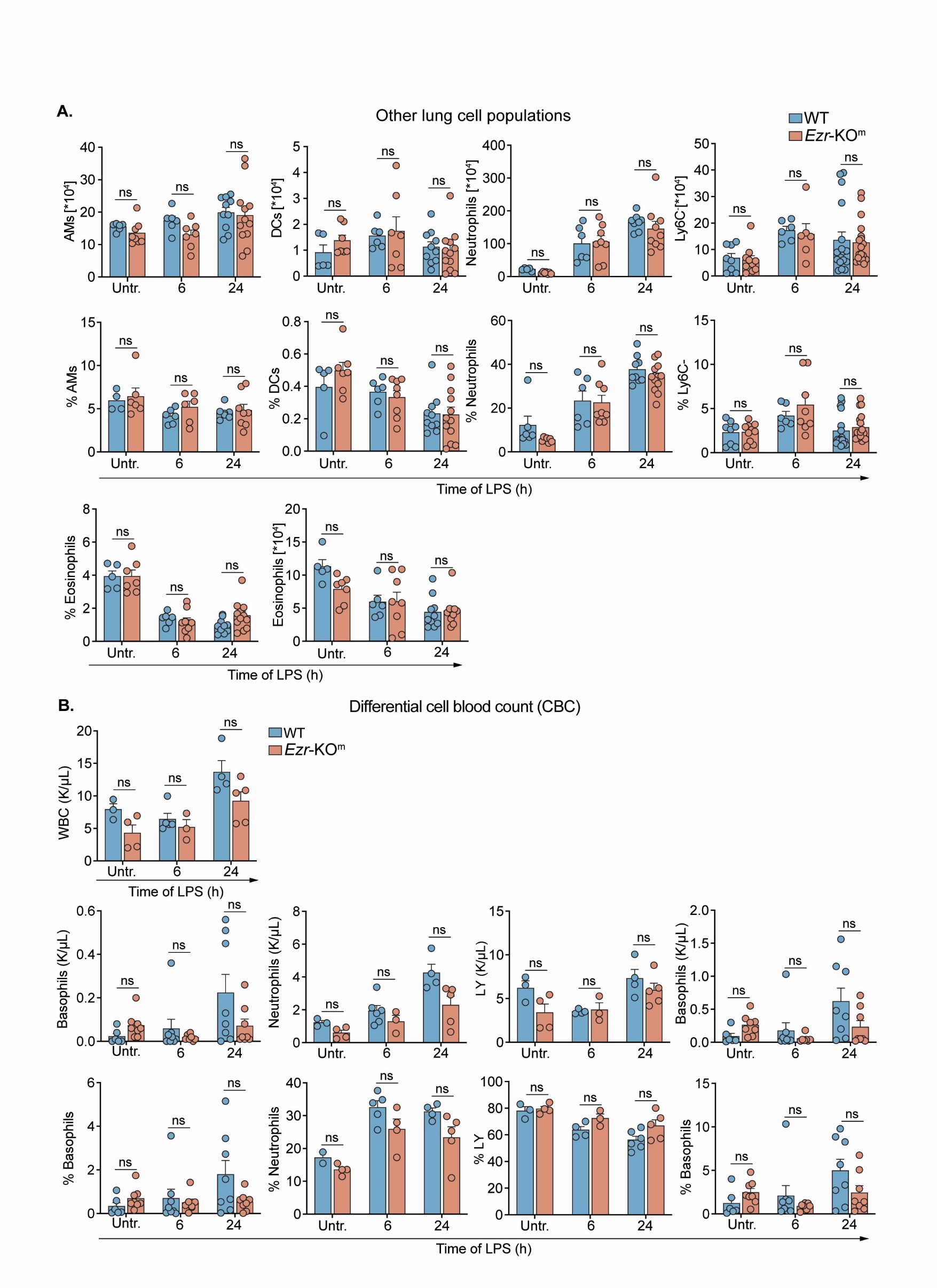
**

**Supplementary Fig. S4**

**Supplementary Fig. S4. Characterization of lung and blood immune cells in WT and *Ezr*-KO^m^ mice at steady state and in response to LPS.**

(A) Flow cytometry analysis showing the total cell numbers and percentages of alveolar macrophages (AMs), dendritic cells (DCs), neutrophils and Ly6C^-^ monocytes among CD45+ cells in the lung inferior lobe of untreated and LPS treated (6h and 24h post LPS nebulization) WT and *Ezr*-KO^m^ mice. Total cell numbers = % of viable cells multiplied by lung cell count in the inferior lung lobe. (B) Differential cell blood counts (CBC) of white blood cells (WBC), eosinophils, neutrophils, lymphocytes (LY), and basophils in untreated and LPS-treated (6h and 24h) WT and *Ezr*-KO^m^ mice. Data were generated from three independent experiments with n=3-6 mice per genotype and time point. Biological replicates are represented by a dot. Bars are depicted as means ± SEM, significance was tested by One-way ANOVA and Tukey’s multiple comparisons test between genotypes for each time point separately (ns - non significant). Related to Fig. 2.


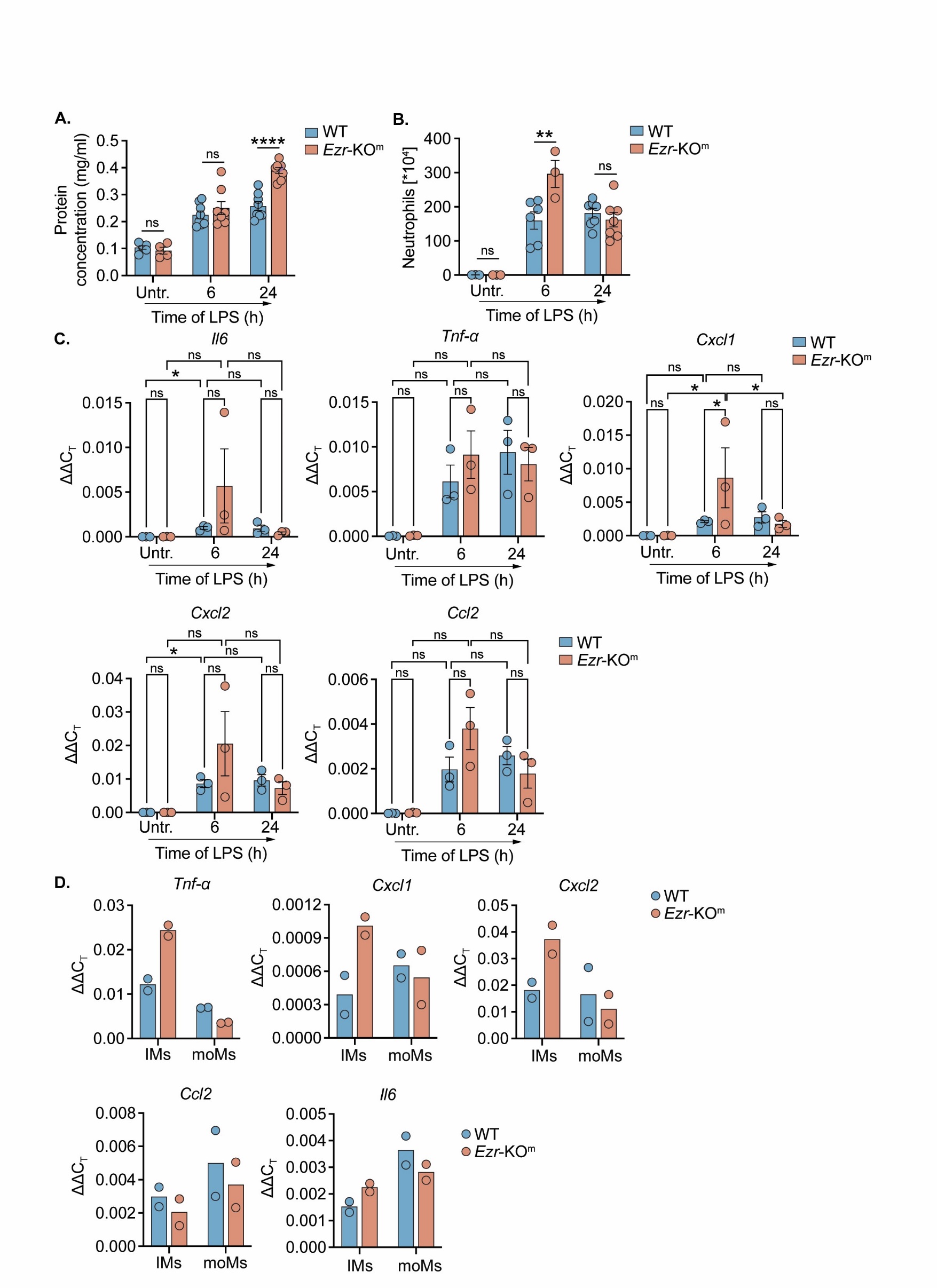


**Supplementary Fig. S5**

**Supplementary Fig. S5. *Ezr*-KO^m^ mouse have a temporary increased lung inflammation in response to LPS compared to controls.**

WT and *Ezr*-KO^m^ mice were treated/untreated (Untr.) with LPS, and the bronchoalveolar fluid (BALF) supernatants and lung tissues were collected at different time points (Untr., 6h and 24h) post-stimulation. (A) Bradford assay showing the total protein concentration in the BALF supernatants of WT and *Ezr*-KO^m^ mice. (B) Flow cytometry analysis showing neutrophil number in the BALF of WT and *Ezr*-KO^m^ mice at different time points post LPS stimulation. (C) Transcriptional expression levels of pro-inflammatory cytokines (*IL-6*, *TNFα) and chemokines* (*Cxcl1*, *Cxcl2* and *Ccl2*) in the lung tissues of WT and *Ezr*-KO^m^ mice at the time indicated. (D) Transcriptional expression levels of *TNFα*, *Cxcl1, Cxcl2, Ccl2* and *IL-6* in the lung-sorted interstitial macrophages (IMs) and monocyte-derived MΦs (moMs) of WT and *Ezr*-KO^m^ mice. Lung-sorted cells were pooled from 3 mice per genotype. Dots in each graph represent technical replicate values obtained from the pooled cells. Data were generated from three independent experiments. Only for (D), lung-sorted cells were pooled from 3 mice. Bars are depicted as means ± SEM, significance was tested by One-way ANOVA and Tukey’s multiple comparisons test between genotypes for each time point separately (* p<0.05; ** p<0.005, **** p<0.0001). Related to Fig. 2.

**
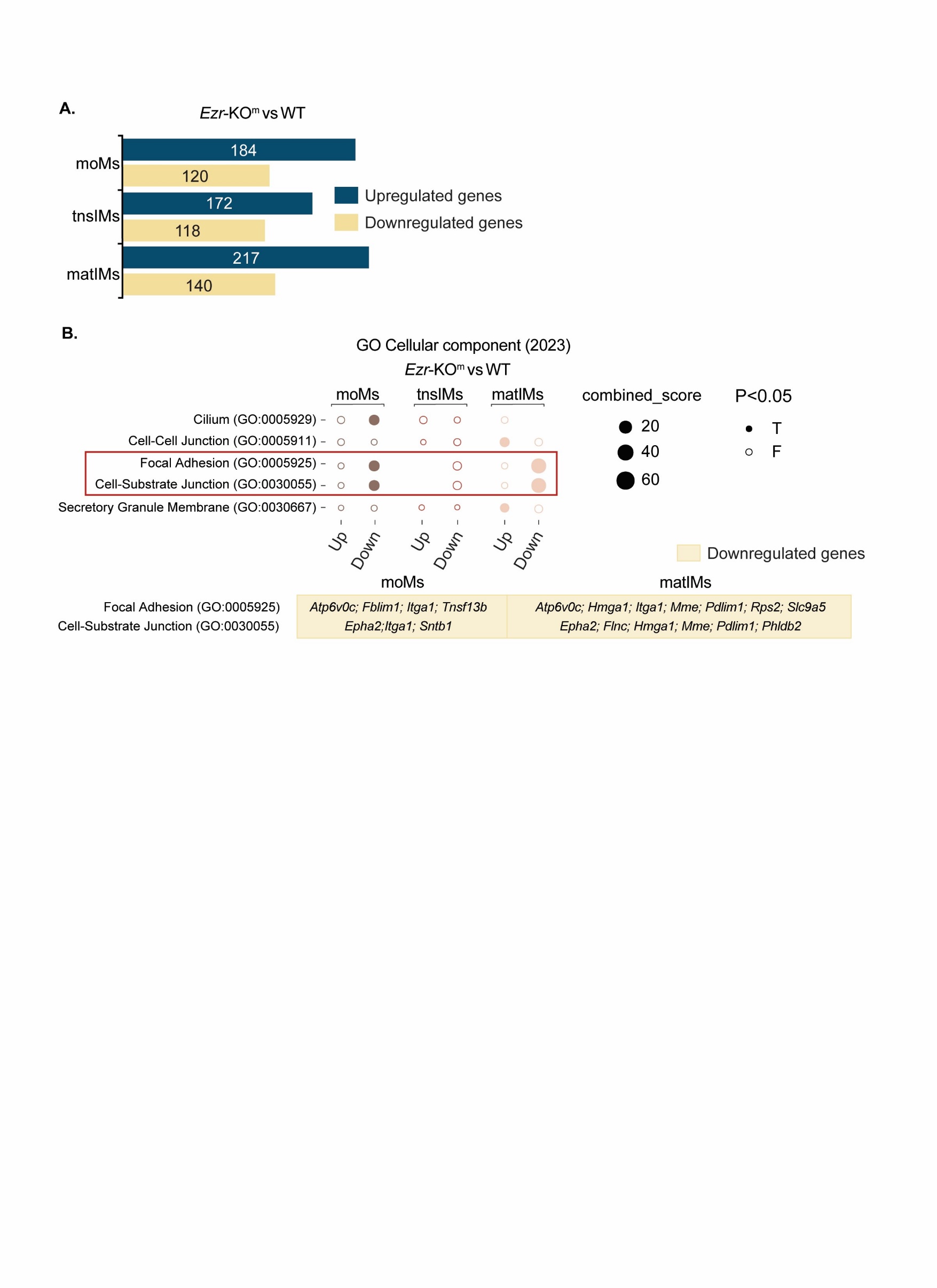
**

**Supplementary Fig. S6**

**Supplementary Fig. S6.** **Characterization of lung macrophage gene expressions, differentially expressed genes (DEGs) and enrichment pathways in WT and *Ezr*-KO^m^ mice in response to LPS.**

(A) Number of upregulated (blue) and downregulated (yellow) DEGs between *Ezr*-KO^m^ and WT monocyte derived macrophages (moMs), transitional interstitial macrophages (tnsIMs), and mature interstitial macrophages (matIMs) populations after LPS stimulation. (B) Gene Ontology (GO) cellular component enrichment analysis of differentially expressed pathways and genes in *Ezr*-KO^m^ vs WT moMs, tnsIMs and matIMs populations treated with LPS. Node size: odds ratio associated with enrichment; p-value for Fisher’s exact test. Data were generated from three mice per genotype. The significance for RNA-seq data was tested with edgeR, as detailed in the methods. Related to Fig. 4.

**
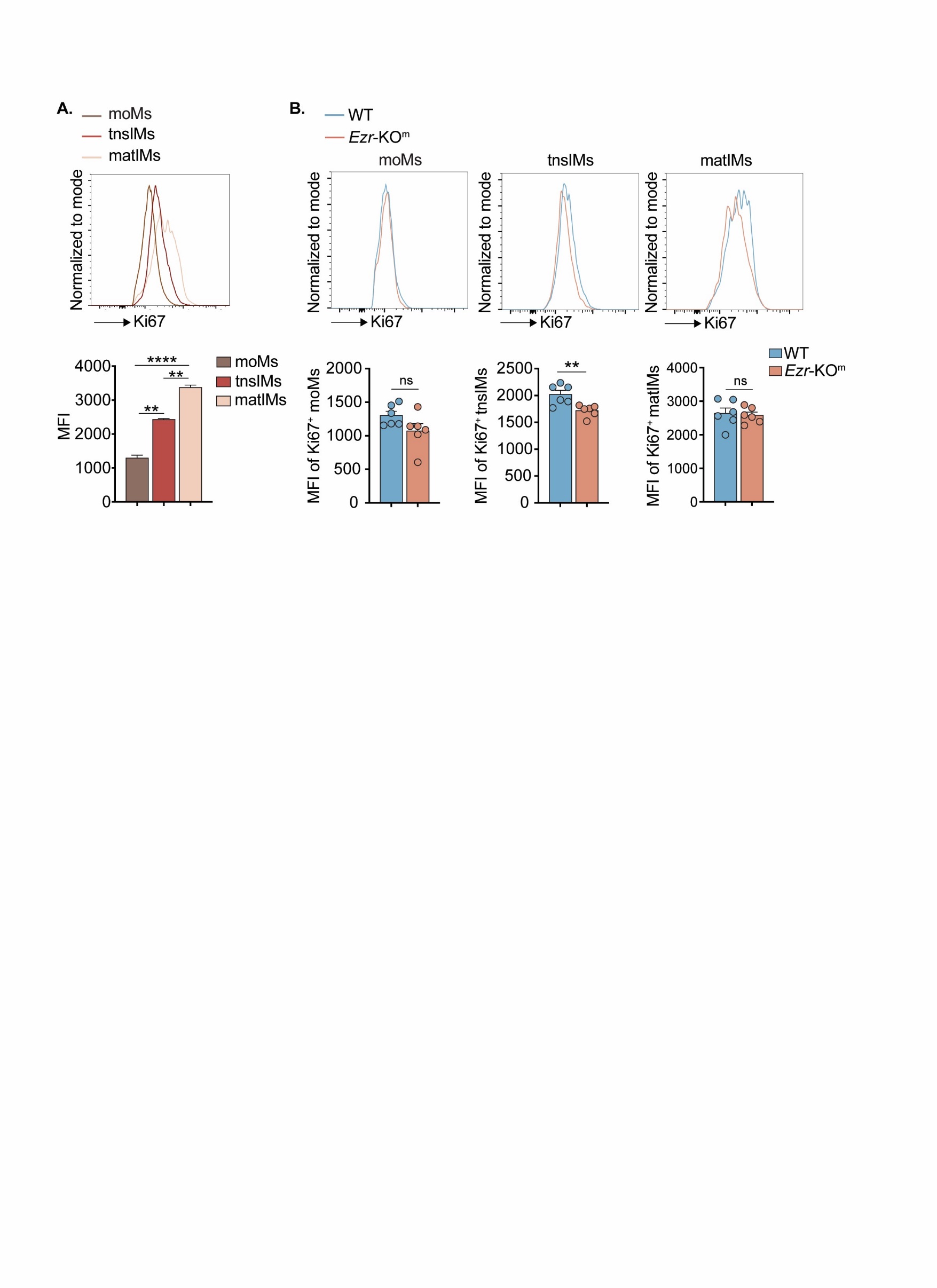
**

**Supplementary Fig. S7**

**Supplementary Fig. S7. Monocyte/MΦs lacking ezrin exhibit proliferation defects in response to LPS.**

(A) Flow cytometry analyzed histogram profiles depicting the upward shift of the Ki67 expression within BrdU^+^Ki67^+^ populations in monocyte derived macrophages (moMs), transitional interstitial macrophages (tnsIMs), and mature interstitial macrophages (matIMs) of LPS-treated WT mice, and the bar graph depicts their corresponding mean fluorescence intensities (MFI). (B) Histogram profiles depicting the downward shift of the Ki67 expression within BrdU^+^Ki67^+^ moMs, tnsIMs and matIMs in *Ezr*-KO^m^ compared to WT mice. Quantification of Ki67 MFIs within BrdU^+^Ki67^+^ moMs, tnsIMs and matIMs between *Ezr*-KO^m^ and WT mice. Data are represented as mean ± SEM from two independent experiments with three or more mice per genotype per experiment. Each dot represents a biological replicate. Statistical analysis was performed using one-way ANOVA or Tukey’s test for multiple comparisons between the genotype and treatment conditions. **p < 0.01, ****p < 0.0001 and ns – non-significant. Related to Fig. 5.

**
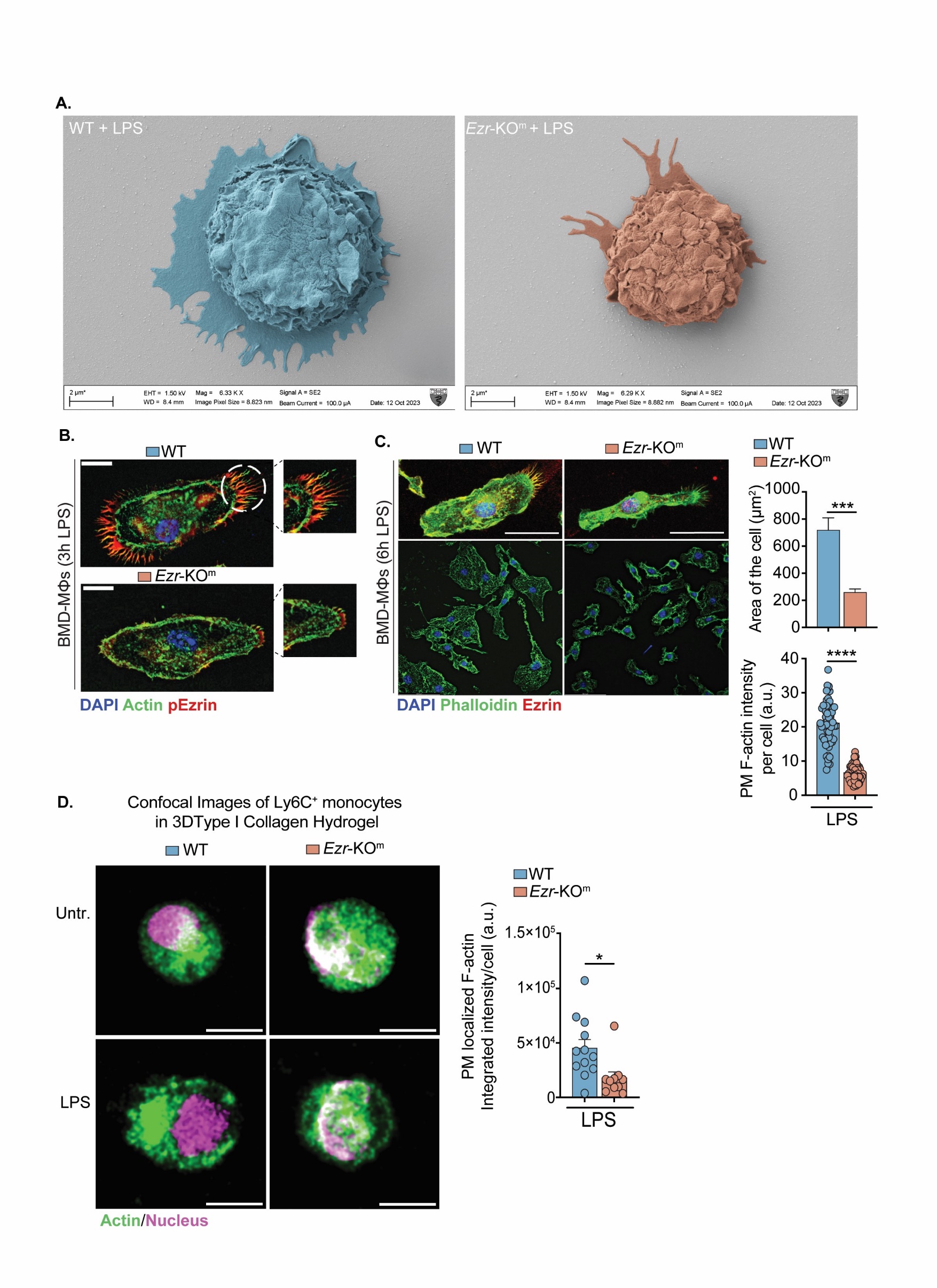
**

**Supplementary Fig. S8**

**Supplementary Fig. S8. Ezrin is required for efficient monocyte/MΦ filipodia formation and cell spreading during activation with LPS.**

(A) Representative scanning electron microscope (SEM) images of LPS-treated Ly6C^+^ monocytes showing altered filopodia formation and impaired cell spreading in *Ezr*-KO^m^ mice compared to WT mice. Cells were cultured on a type I collagen-coated surface. Scale bar = 2 μm. (B-C) Bone marrow derived (BMD)-MΦs were cultured from murine WT and *Ezr*-KO^m^ and treated with LPS for 3h (B) and 6h (C) on a collagen-coated surface. Representative immunofluorescence (IF) images showing murine WT and *Ezr*-KO^m^ BMD-MΦs treated with LPS for 3h (B) and 6h (C). (C) Quantification of the area of the cells showing the reduced cellular area in *Ezr*-KO^m^ BMD-MΦs (area: 258 ± 63 μm^2^) compared to WT BMD-MΦs (area: 756 ± 121 μm^2^) and plasma membrane (PM) filamentous (F)-actin intensity per cell, on the type I collagen surface in response to LPS. (D) Representative 3-dimensional (3D) confocal fluorescence microscope images of Ly6C^+^ monocytes in 3D type I collagen hydrogel from WT and *Ezr*-KO^m^ mice, untreated or treated with LPS, showing actin staining (green) and nucleus (pink). In addition, the graph depicts the quantification of PM localized F-actin intensity from WT and *Ezr*-KO^m^ monocytes. Scare bar = 5 μm. Data are represented as mean ± SEM from two independent experiments. Statistical analysis was performed using students’ t-test for multiple comparisons between the genotype and treatment conditions. (*p < 0.05 and ***p < 0.001). Related to Fig. 6.


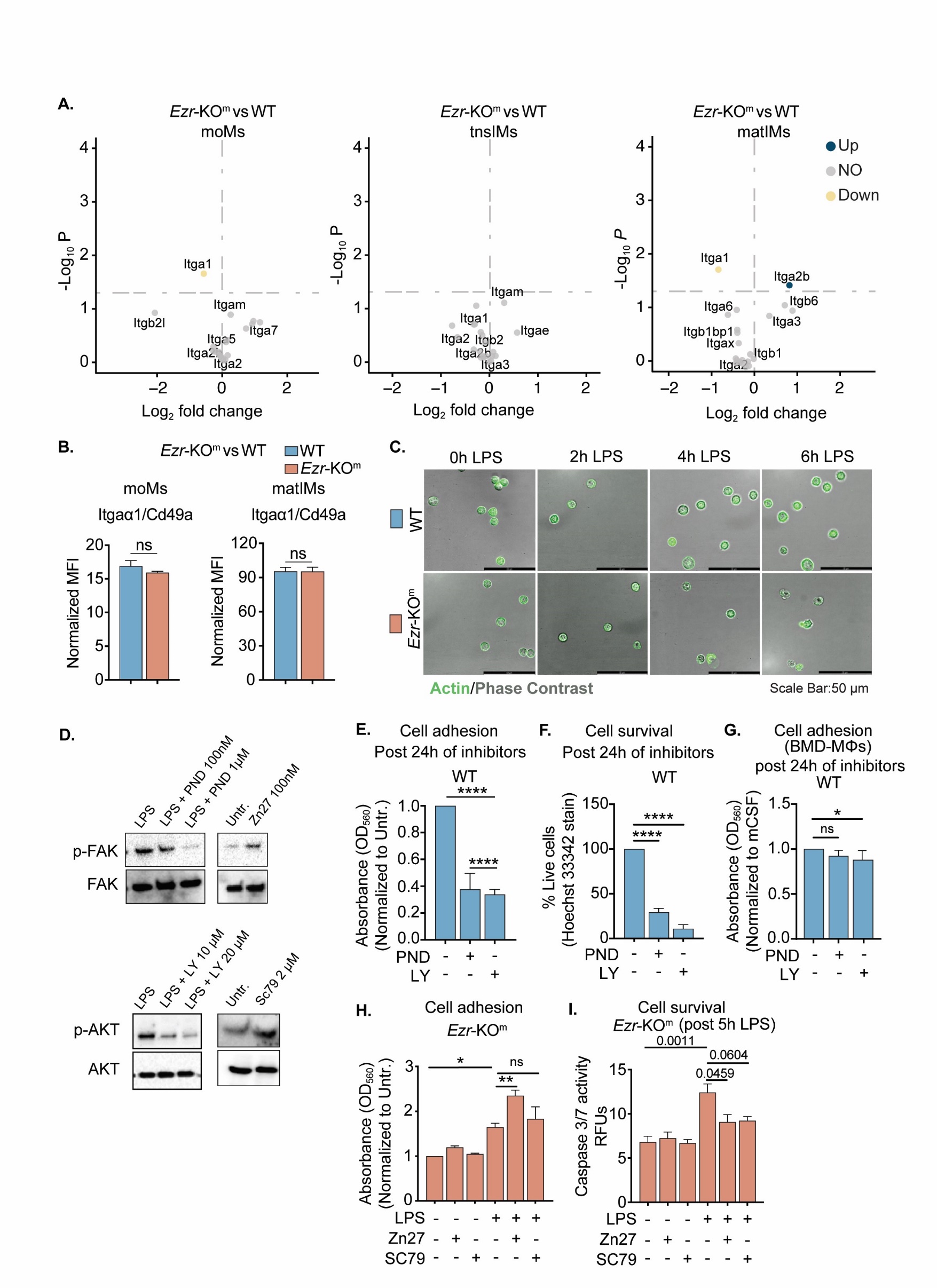


**Supplementary Fig. S9**

**Supplementary Fig. S9.** **Loss of ezrin does not alter monocytes/MΦ integrin expressions but alters downstream FAK/AKT signaling pathways in Ly6C^+^ monocytes in response to LPS.**

(A) Volcano plots of bulk RNA seq characterized log2 fold change of different integrin subunits levels in monocyte derived macrophages (moMs), transitional interstitial macrophages (tnsIMs), and mature interstitial macrophages (matIMs) of *Ezr*-KO^m^ mice compared to WT. (B) Quantification of integrin α1 expression in lung moMs and matIMs of WT and *Ezr*-KO^m^ mice treated with LPS, as assessed by flow cytometry. (C) Representative images showing Ly6C^+^ monocyte actin (green) staining and their altered morphology in *Ezr*-KO^m^ mice compared to WT in a time-dependent response to LPS. Scale bar = 50 μm. (D) Representative WB of phospho-FAK (pFAK, tyrosine 397), total FAK, phospho-AKT (pAKT, serine 473) and total AKT in murine WT BMD-MΦs. The cells were pretreated with/without PND-1186 (100 nM, 1 μM; FAK inhibitor, shown as PND), Zn27 (100 nM; FAK activator), LY294002 (10 μM, 20 μM; AKT inhibitor, shown as LY) and Sc79 (2 μM; AKT activator) for 30 min and later treated/untreated with LPS (1 μg/ml, 30 min). (E-G) Primary bone marrow (BM) monocytes or bone marrow derived-MΦs (BMDMs) isolated or differentiated from WT mice were cultured on a collagen-coated surface in the presence of m-CSF (20 ng/mL) and untreated/treated with DMSO (vehicle), PND-1186 (1 μM), or LY294002 (10 μM) for 24h. (E) Quantification of crystal violet absorbance emitted from adherent primary WT BM monocytes. (F) Quantification of Hoechst stained cell numbers of primary WT BM monocytes. (G) Quantification of crystal violet absorbance emitted from adherent WT BMD-MΦs. All data were normalized to untreated (Untr.). (H-I) Primary BM monocytes from *Ezr*-KO^m^ mice were cultured on a collagen-coated surface and untreated or treated with LPS (1 ug/mL) and/or DMSO (vehicle), Zn27 (100 nM) or Sc79 (2 μM) for up to 6h. (H) Quantification of crystal violet absorbance emitted from adherent primary WT BM monocytes post 6h of treatment (I) Relative fluorescence units measuring caspase3/7 activity in primary *Ezr*-KO^m^  BM monocytes 5h post-treatment. (A-B) Data are represented from n = 3 mice. (C) Representative image form one experiment (quantified in Fig. 7D). Bars are depicted as mean ± SEM from two or three independent experiments. (E-I) Data are represented from one experiment with n = 3-4 biological replicates per genotype. Bars are depicted as mean ± SEM. Statistical analysis was performed using a Student’s t-test between the genotypes. **p < 0.01, ****p < 0.0001 and ns – non-significant. Related to Fig. 7.

**SUPPLEMENTARY VIDEOS:**

**Video 1:** Representative live imaging of WT monocytes in response to LPS for 6h. Images were captured continuously for 6h at 30-minute intervals. Yellow channel depicts actin stain and brightfield channel depicts cell morphology.

**Video 2:** Representative live imaging of Ezr-KO^m^ monocytes in response to LPS for 6h. Images were captured continuously for 6h at 30-minute intervals. Yellow channel depicts actin stain and brightfield channel depicts cell morphology.

**SUPPLEMENTARY MATERIALS AND REAGENTS:**

Antibodies used for flow cytometry are as follows: Rat anti mouse CD45 BUV395 clone 30-F11 (Cat# 565967, BD), Rat anti mouse CD11b PE-Cy7 clone M1/70 (Cat# 25-0112-82, Invitrogen), Rat anti mouse CD64 APC clone X54-5/7.1 (Cat#139306, BioLegend), Rat anti mouse Ly6C clone AL-21 (Cat# 563011, BD), Rat anti mouse CD11c BV711 clone HL3 (Cat# 563048, BD), Rat anti mouse Ly6G AF700 clone 1A8 (Cat# 551459, BD), Rat anti mouse Siglec-F PerCP-Cy5.5 clone E50-2440 (Cat# 565526, BD), Rat anti mouse I-A/I-E (MHC-II) APC/Fire 750 clone M5/114.15.2 (Cat# 107652, BioLegend), Rat anti mouse Siglec-F PerCP-Cy5.5 clone E50-2440 (Cat# 565526, BD), Rat anti mouse I-A/I-E (MHC-II) APC/Fire 750 clone M5/114.15.2 (Cat# 107652, BioLegend), FcBlock (Cat# 553141, BD), LIVE/DEAD™ Fixable Aqua Dead Cell Stain (Cat#L34957, ThermoFisher Scientific).

Antibodies used for western blot and/or immunofluorescence are as follows: Mouse-HRP anti-beta actin (Cat# sc47778, Santa Cruz), Rabbit mAb anti-pezrin (Thr567) (Cat#11202, SAB), Rabbit polyclonalAb anti-ezrin [EP886Y] (Cat#ab40839, Abcam), Rabbit polyclonal anti-moesin (Q480) (Cat#3150, Cell Signaling), Rabbit polyclonal anti-pFAK(Tyr397) (Cat# 3283, Cell Signaling), Rabbit polyclonal anti-FAK (Cat# 3285, Cell Signaling), Rabbit polyclonal anti-pAKT (Ser473) (Cat# 9271, Cell Signaling), Rabbit polyclonal anti-AKT (Cat# 9272, Cell Signaling), Goat anti-rabbit IgG-HRP (Cat# sc-2004, Santa Cruz), AlexaFluor™ 488 phalloidin (Cat#R37110, ThermoFisher Scientific), DAPI solution (Cat#62248, ThermoFisher Scientific), Rat mAb anti-CD68 [FA-11] (Cat#ab53444, Abcam), Rabbit envision secondary antibody (Cat# K4001-8, Agilent/Dako), Goat HRP-anti-Rabbit (Cat#sc-2030, SantaCruz), Donkey anti-Rat IgG (H+L) Highly Cross-Adsorbed Secondary Antibody, Alexa Fluor™ 594 (Cat#A-21209, Invitrogen), Donkey anti-rabbit IgG Secondary Ab, 555 (Cat#A21572, Mol Probe).

Commercial chemicals, small molecule inhibitors, peptides, and recombinant proteins are as follows: PND 1186 - FAK inhibitor (Cat#6891, Tocris), LY294002 - AKT inhibitor (Cat#1130, Tocris), Zn27 - FAK activator (Cat#HY-134570, MedChemExpress), Sc79 - AKT activator (Cat#HY-18749, MedChemExpress), Hoechst 33342 (Cat#ab228551, Abcam), Bovine Serum Albumin (Cat#A9647, Millipore Sigma), cOmplete Mini EDTA-free protease inhibitor cocktail (Cat# 11836170001, Roche), PBS (Cat# 10010023, Gibco), UltraPure Low Melting Point agarose (Cat# 16520-050, Invitrogen), lipopolysaccharides from *Pseudomonas aeruginosa* (Cat# L9143, Millipore Sigma), Cell Lysis Buffer (Cat# 9803, Cell Signaling), PhosSTOP (Cat# 4906837001, Roche), 4-15% Mini PROTEAN TGX Gels (Cat# 4561086, Bio-Rad), ECL Plus Western blotting system (Cat# RPN2132, GE Healthcare), SuperScript II Reverse Transcriptase (Cat# 18064-022, ThermoFisher), RBC lysis buffer (Cat# 00-4300-54, eBioscience), PhosSTOP (Cat# 4906837001, Roche), 4-15% Mini PROTEAN TGX Gels (Cat# 4561086, Bio-Rad), Bio-Rad Protein assay reagent concentrate (Cat#5000006, BioRad).

Commercial kits: Lung dissociation kit (mouse) (Cat# 130-095-927, Mitenyi Biotec), miRNEasy Micro kit (Cat# 217084, Qiagen), Agilent RNA6000 Pico Kit (Cat# G2938-90046, Agilent), SMART-Seq v4 Ultra Low Input RNA Kit for Sequencing (Cat# 634890, Takara Bio), Nextera XT DNA Sample Preparation kit (Cat# FC-131-1096, Illumina), miScript II RT kit (Cat# 218161, Qiagen), Monocyte Isolation Kit (mouse) (Cat#130-100-629, Mitenyi Biotec).

Experimental models used are as follows: WT mice B6.*Cx3cr1*^tm1.1(cre)Jung/J^ (Stock No. 025524, Jackson Laboratory), *Ezr*-KO^m^ mice B6.*Ez*^fl/fl^ [1,2]-*Cx3cr1*^tm1.1(cre)Jung/J^ - in collaboration with Dr. Neetu Gupta, *CCR2* knockout mice B6.129S4-*Ccr2*^tm1Ifc/J^ (Stock No. 004999, Jackson Laboratory) [3], *Cx3cr1*gfp mice B6.129P2(Cg)-*Cx3cr1*^tm1Litt/J^ (Stock No. 005582, Jackson Laboratory) [4].

Software and algorithms used are as follows: FACSDiva (BD), FlowJo (BD), Prism (GraphPad), enrichR package (<https://maayanlab.cloud/Enrichr>), edgeR package (<https://bioconductor.org>), Qlucore (<https://qlucore.com/>), MetaCore (<https://portal.genego.com/>), RStudio (<https://rstudio-education.github.io/hopr/>), FIJI (ImageJ) (<https://imagej.net/software/fiji/>), LAS X Microscope Software (<https://www.leica-microsystems.com/>). In addition, we used a Nebulizer Pulmo-Aide Compressor (Cat# DRV5650D, Natallergy) for nebulizing the mouse.

**SUPPLEMENTARY REFERENCES:**

1. Pore D, Gupta N. The ezrin-radixin-moesin family of proteins in the regulation of B-cell immune response. Crit Rev Immunol. 2015;35(1):15-31.

2. Saotome I, Curto M, McClatchey AI. Ezrin is essential for epithelial organization and villus morphogenesis in the developing intestine. Dev Cell. 2004;6(6):855-64.

3. Boring L, Gosling J, Chensue SW, Kunkel SL, Farese RV, Jr., Broxmeyer HE, et al. Impaired monocyte migration and reduced type 1 (Th1) cytokine responses in C-C chemokine receptor 2 knockout mice. J Clin Invest. 1997;100(10):2552-61.

4. Jung S, Aliberti J, Graemmel P, Sunshine MJ, Kreutzberg GW, Sher A, et al. Analysis of fractalkine receptor CX(3)CR1 function by targeted deletion and green fluorescent protein reporter gene insertion. Mol Cell Biol. 2000;20(11):4106-14.
