# Supplementary material for: Ezrin drives adaptation of monocytes to the inflamed lung microenvironment
Source: Cell Death Dis. 2024 Nov 29;15(11):864. doi: 10.1038/s41419-024-07255-8 (PMC11607083; doi:10.1038/s41419-024-07255-8)
Supplement: Supplementary file 2 — Uncropped western blots [file 41419_2024_7255_MOESM2_ESM.pdf]

Figure 1C

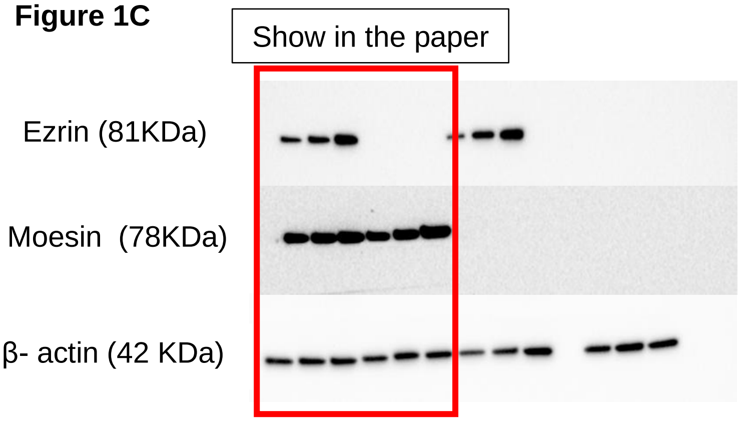

Figure 1D

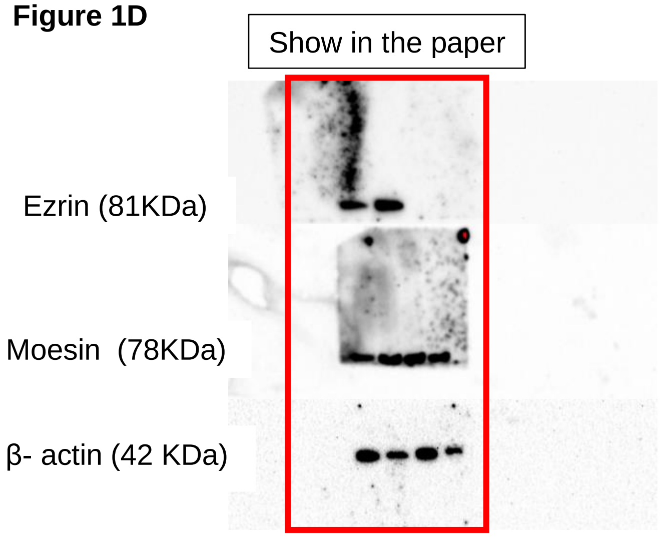

Figure 7C

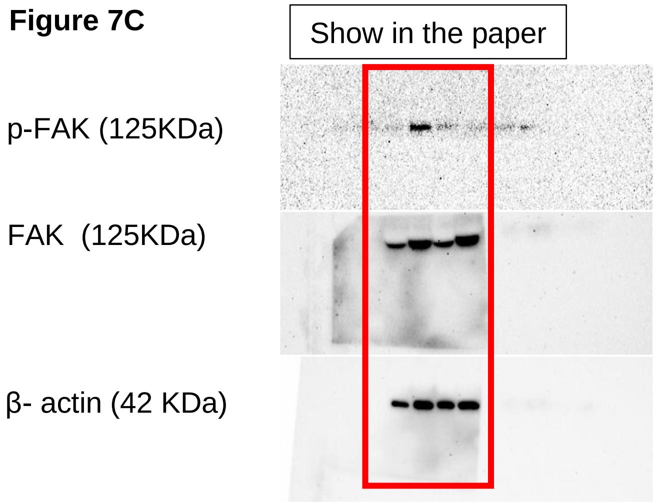

Figure 7C

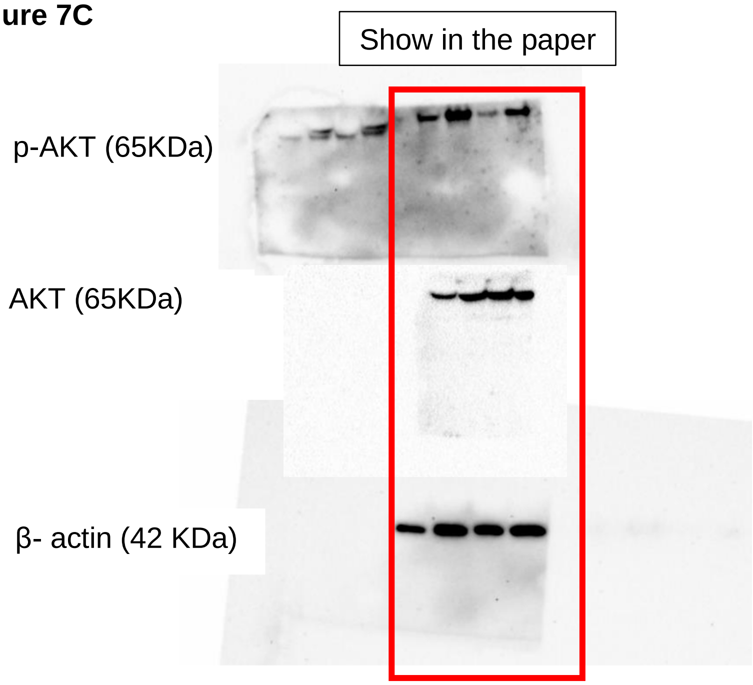

Figure S9D

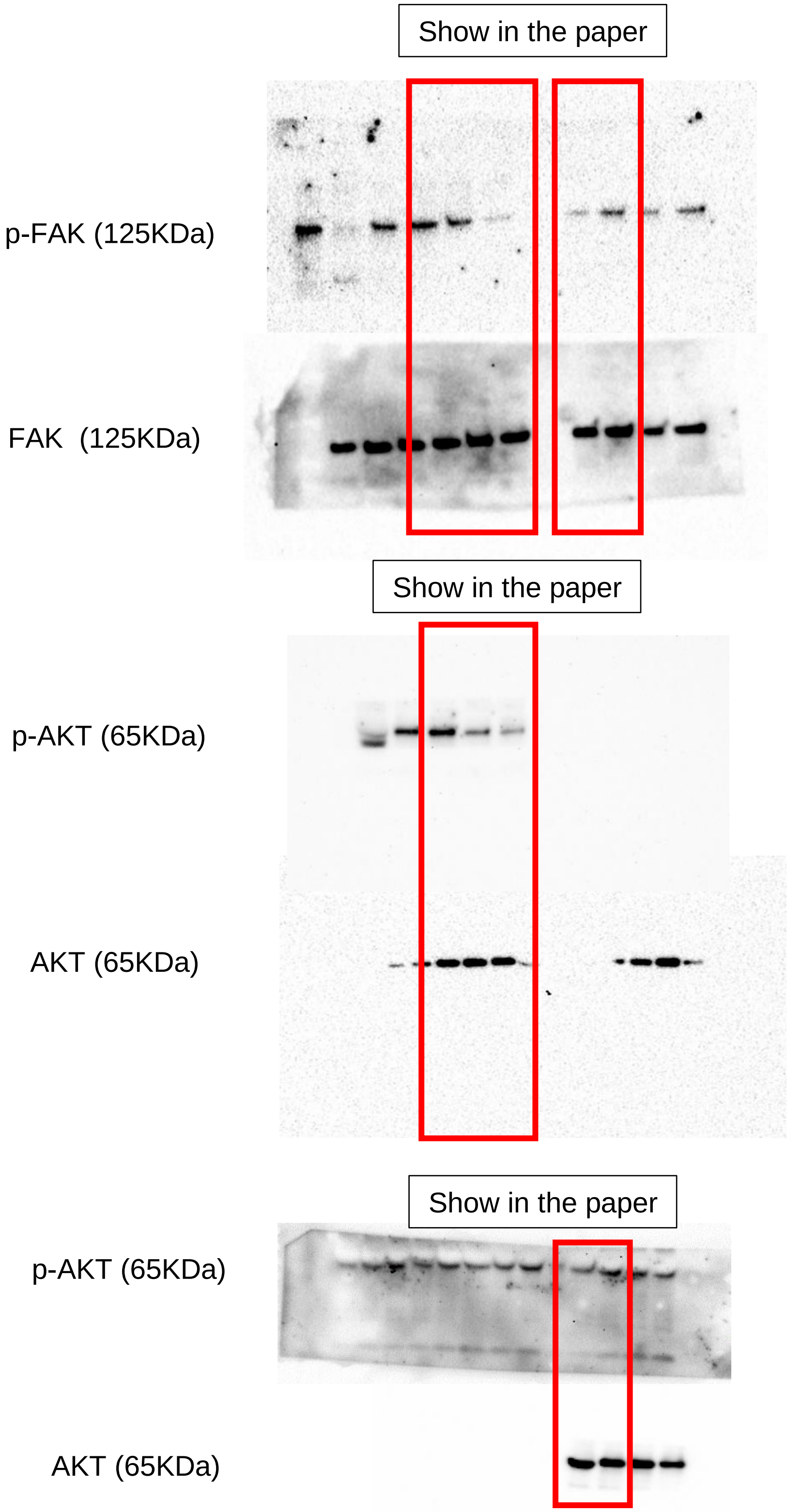
Show in the paper
p-FAK (125KDa)
FAK (125KDa)
Show in the paper
p-AKT (65KDa)
AKT (65KDa)
Show in the paper
p-AKT (65KDa)
AKT (65KDa)
